# Modulation of the immune response by the host defense peptide IDR-1002 in chicken hepatic cell culture

**DOI:** 10.1038/s41598-023-41707-z

**Published:** 2023-09-04

**Authors:** Csilla Sebők, Patrik Tráj, Máté Mackei, Rege Anna Márton, Júlia Vörösházi, Ágnes Kemény, Zsuzsanna Neogrády, Gábor Mátis

**Affiliations:** 1 Division of Biochemistry, Department of Physiology and Biochemistry, University of Veterinary Medicine, István utca 2, 1078 Budapest, Hungary; 2 Department of Pharmacology and Pharmacotherapy, Faculty of Medicine, University of Pécs, Szigeti u. 12, 7624 Pécs, Hungary; 3 Department of Medical Biology, Faculty of Medicine, University of Pécs, Szigeti u. 12, 7624 Pécs, Hungary

**Keywords:** Peptides, Cell death and immune response, Chemokines, Chemokines, Interferons, Interleukins, Inflammation, Monocytes and macrophages, Antimicrobial responses

## Abstract

IDR-1002, a synthetic host defense peptide (HDP), appears to be a potential candidate for the treatment of bacterial infections and the consequent inflammatory response due to its potent immunomodulatory activity. This is of relevance to the emerging issue of antimicrobial resistance in the farming sector. In this study, the effects of IDR-1002 were investigated on a chicken hepatocyte‒non-parenchymal cell co-culture, and the results revealed that IDR-1002 had complex effects on the regulation of the hepatic innate immunity. IDR-1002 increased the levels of both RANTES (Regulated on Activation, Normal T cell Expressed and Secreted) and Macrophage colony stimulating factor (M-CSF), suggesting the peptide plays a role in the modulation of macrophage differentiation, also reflected by the reduced concentrations of interleukin (IL)-6 and IL-10. The pro-inflammatory cytokine release triggered by the bacterial cell wall component lipoteichoic acid (LTA) was ameliorated by the concomitantly applied IDR-1002 based on the levels of IL-6, chicken chemotactic and angiogenic factor (CXCLi2) and interferon (IFN)-γ. Moreover, the production of nuclear factor erythroid 2-related factor 2 (Nrf2), an essential transcription factor in the antioxidant defense pathway, was increased after IDR-1002 exposure, while protein carbonyl (PC) levels were also elevated. These findings suggest that IDR-1002 affects the interplay of the cellular immune response and redox homeostasis, thus the peptide represents a promising tool in the treatment of bacterially induced inflammation in chickens.

## Introduction

Large-scale poultry farming has been expanding rapidly since the 1970s, and the growing demand for eggs and meat is presenting the sector with increasing challenges^1^. Enteric bacterial infections represent a constant and complex problem in the poultry industry, as they can lead to reduced productivity or even an increase in mortality rates^2^. The use of prophylactic antibiotics is no longer permitted within the European Union^3^ but, it is often not feasible to treat such infections at herd level without their application. Growing antimicrobial resistance is making this increasingly difficult, however, not only because of treatment failure, but also because more and more antibiotics are being restricted due to their importance for human health^4^. For this reason, it is of vital importance to develop alternative therapies that can complement or replace antibiotics.

Endotoxins released during bacterial infections (such as lipopolysaccharide [LPS] from Gram-negative, and lipoteichoic acid [LTA] from Gram-positive bacteria), especially when the integrity of the intestinal wall is compromised, are transported from the intestinal tract to the liver via the portal circulation^5^. The liver plays a particularly important role in innate immunity by eliminating pathogens that enter the circulation from the gut and by influencing the systemic immune response with its pattern recognition receptors (PRRs)^6^. Kupffer cells, as resident macrophages of the liver are the first to interact with harmful toxins and pathogens, therefore they have an essential role in phagocytosis, antigen presentation, and cytokine production^7^. LTA as a pathogen-associated molecular pattern acts as a CD14 and TLR2 (toll-like receptor) agonist, therefore it can activate cytokine production through the NF-κB (nuclear factor kappa light chain enhancer of activated B cells) pathway^8^.

Host defense peptides (HDPs), also called antimicrobial peptides (AMPs), are promising candidates for the therapy of infectious and inflammatory diseases, therefore they may be a useful tool in the reduction of antibiotic usage. More than 2600 of these immunologically active small cationic peptides have been described, including both peptides of natural (produced as part of the innate immune system of almost all organisms) and synthetic origin^9^. Initially, these molecules gained attention for their direct antimicrobial activity (hence the name AMP), but later it was proven that this effect can be inhibited by physiological concentration of salts, glycosaminoglycans and proteins^10^. In recent decades, HDPs were recognized as an essential part of the innate immune system as they can stimulate the defense mechanisms considerably^11^. Since then, they have gained attention by virtue of their endotoxin binding, chemotactic and immunostimulant effects. HDPs were also found effective against bacterial, viral, autoimmune, and cancerous diseases, as well as in stimulating wound healing^11–14^.

Although several studies have indicated HDPs to be very promising candidates for the treatment of various infectious, inflammatory, and even immunological diseases, there are still some obstacles that make it challenging to apply them in practice. Some of those factors are the potential toxicity, the susceptibility to proteases, and the cost of production. To reduce costs and the probability of proteolysis, new production techniques, and shorter peptides should be developed^15^. Innate Defense Regulator-1002 (IDR-1002), with a length of 12 amino acids, could be advantageous in the latter aspect. In recent years, several HDPs have reached the clinical stage of drug development for intravenous, topical, and oral use^15^, but, mainly due to high costs, the progress is more prominent in human medicine. To the authors' knowledge, no relevant progress has been made so far in the veterinary field, and it is therefore particularly important to obtain more detailed information on promising individual peptides, thus facilitating possible future trials. In poultry, their use as feed additives is the main focus, primarily for practical reasons^16^. Since the European Union has banned the use of antibiotics as growth promoters^17^, there has been a continuous search for candidates that can both improve growth parameters and help to prevent and combat various flock-level diseases. IDR-1002 offers various benefits over other HDPs, as the peptide is non-toxic to cells and due to regulating the immune system rather than directly killing bacteria, far smaller dosages may be enough to elicit a beneficial effect^18^.

The application of natural HDPs has proven to be difficult due to their unfavorable characteristics, such as their hemolytic activity, cytotoxicity against mammalian cells, stimulation of mast cell degranulation, and promotion of apoptosis^19^. In 2007, a derivative of bactenecin (which is one of the natural HDPs of cattle), called innate defense regulator peptide (IDR-1), was studied and declared as a candidate without the typical adverse effects of the group. Although its direct antimicrobial activity was not observed in vitro, it was shown to be effective in protecting against infections (including several pathogenic bacteria) in a mouse model. IDR-1 was found to significantly modulate the gene expression of human monocytes, which can lead to the induction of several cytokines and chemokines, indicating that it can enhance the natural immune response^20^.

IDR-1002 belongs to the group of innate defense regulators (IDRs), and previous studies suggest an outstanding ability to modulate cytokine production and to reduce the inflammatory response induced by LPS, while demonstrating no signs of cytotoxicity up to much higher concentrations than the human cathelicidin LL-37^19,21,22^. Although IDR-1002 has substantially lower direct antibacterial activity than other naturally generated antimicrobial peptides, evidence supports that this peptide is effective against infections caused by several pathogenic bacteria, and that it simultaneously decreases pathogen-induced inflammation. For example, IDR-1002 has been demonstrated to be effective in the treatment of *Escherichia coli* and *Staphylococcus aureus* infections^19,23^, as well as in the treatment of pneumonia caused by *Pseudomonas aeruginosa* in mouse models^24,25^. In addition, promising results were obtained recently from studies examining the effect of IDR-1002 on dental pulp regeneration therapies^26,27^.

In the present study, the effects of IDR-1002 were investigated on a primary hepatocyte–non-parenchymal cell co-culture of chicken origin exposed to *Staphylococcus aureus* derived LTA. Our aim was to explore the effect of the peptide on the viability of the cells and on the hepatic immune system in the presence and absence of LTA-induced inflammation by measuring pro-inflammatory cytokines such as chicken chemotactic and angiogenic factor (CXCLi2, also known as chicken interleukin [IL]-8), IL-6, IL-16, interferon (IFN)-γ, the anti-inflammatory cytokine IL-10, and signaling molecules that influence macrophage differentiation and function, like macrophage colony stimulating factor (M-CSF) and RANTES (Regulated on Activation, Normal T cell Expressed and Secreted). Furthermore, the oxidative status of the cells was examined by monitoring the extracellular hydrogen peroxide concentration, the intracellular protein carbonyl (PC) levels, which indicate protein damage due to oxidative stress, and the abundance of Nrf2 (nuclear factor erythroid 2-related factor 2) protein, which is an important factor in the antioxidant defense system. Our results provide further insight into the effects of the peptide on a cellular level, thus advancing the development of potential therapeutics for future application in the veterinary medicine.

## Materials and methods

### Isolation of cells

Hepatocytes and non-parenchymal cells were isolated from the liver of a 3-week-old male Ross-308 broiler chicken. The animal was fed according to Ross Technology and all efforts were made to support the welfare of the animal. All experiments were in accordance with European Union laws, institutional guidelines, confirmed by the Local Animal Welfare Committee of the University of Veterinary Medicine Budapest, and permitted by the Government Office (number of permission: GK-419/2020; approval date: 11 May 2020). The study was conducted following the ARRIVE guidelines 2.0 (https://arriveguidelines.org/). Unless stated otherwise, all chemicals were purchased from Merck (Darmstadt, Germany).

Slaughter of the chicken was done under CO_2_ anaesthesia by decapitation. Then the body cavity was opened and after accessing the liver, the portal system was cannulated through the gastropancreaticoduodenal vein. In order to remove the blood and disrupt the connective tissue of the organ, a three-step perfusion was performed with 150 mL of 0.5 M ethylene glycol tetraacetic acid (EGTA) containing Hanks’ Balanced Salt Solution (HBSS) buffer, 150 mL EGTA-free HBSS and in the final step, 100 mL HBSS supplemented with 100 mg collagenase type IV, 7 mM CaCl_2_ and 7 mM MgCl_2_ (Nordmark, Uetersen, Germany) as described previously by Mackei et al.^28^. After removal of the liver and the disruption of the Glisson’s capsule, the cells were suspended in 50 mL ice cold bovine serum albumin (BSA, 1.25 g) containing HBSS buffer and filtered through three layers of sterile gauze. To prevent cell aggregation, this cell suspension was then kept on ice for 45 min. Following this, the suspension was centrifuged three times at 100×*g* for 3 min. The pellet containing hepatocytes was resuspended in Williams Medium E supplemented with 0.22% NaHCO_3_, 50 mg/mL gentamycin, 0.5 µg/mL amphotericin B, 2 mM glutamine, 4 µg/L dexamethasone, 20 IU/L insulin, and 5% fetal bovine serum (FBS). The FBS was only present in the medium for the first 24 h after seeding.

To guarantee that any remaining hepatocytes, cell debris, and erythrocytes were sedimented, the supernatant containing non-parenchymal cells was centrifuged once more at 350×*g* for 10 min. Thereafter, the supernatant was centrifuged at 800×*g* for 10 min, and the pellet was resuspended in Williams Medium E. Viability of the isolated hepatocytes and non-parenchymal cells was confirmed by trypan blue exclusion test. The cell suspensions were diluted to 8.5 × 10^5^ cells/mL in the hepatocyte-enriched fraction and to 1.5 × 10^5^ cells/mL in the non-parenchymal cell-containing fraction.

Cell cultures were prepared using 24-well and 96-well culture plates (Greiner Bio-One, Frickenhausen, Germany) pre-coated with collagen type I. Hepatocyte and non-parenchymal cell fractions were mixed in a 6:1 ratio prior to plating. On the 24-well plates, seeding quantity was 400 µL/well, while 100 µL/well on the 96-well plates. All cell cultures were incubated at 37 °C with high humidity and 5% CO_2_, and culture medium was changed after 4 h. Confluent monolayers were formed following 24 h of incubation.

### Treatments and sampling

After 24 h culturing, cells were exposed to the following substances for 24 h. IDR-1002 (Isca Biochemicals, Exeter, Devon, UK) was added in concentrations of 10, 30 and 90 µg/mL (IDR-low, IDR-medium and IDR-high, respectively) alone and in combination with 50 µg/mL LTA from *Staphylococcus aureus* (n = 6/group). A stock solution of 1000 µg/mL of IDR-1002 and 5000 µg/mL of LTA was prepared, both dissolved in Williams Medium E. The stock solutions were then diluted with Williams Medium E at concentrations corresponding to the treatment groups. The Control group received only Williams Medium E, without any supplementation. After sampling from the cell culture media of 24-well plates, cells were lysed by applying 50 µL/well Mammalian Protein Extraction Reagent (M-PER) lysis buffer and scraped from the surface. Culture media and cell lysate samples were stored at − 80 °C until further processing. 96-well plates were used for the measurement of the metabolic activity of the cells.

### Cellular measurements

CCK-8 test (Cell Counting Kit-8, Merck Cat.Nr. 96992) was used to assess the metabolic activity of the cells on 96-well plates by measuring the amount of NADH + H^+^ produced in the catabolic pathways. The test was carried out in accordance with the manufacturer's instructions. 10 µL of CCK-8 reagent and 100 µL of fresh Williams Medium E was added to each well of the 96-well plate. A Multiskan GO 3.2 reader (Thermo Fisher Scientific, Waltham, MA, USA) was used to measure the absorbance at 450 nm after incubation at 37 °C for 2 h.

Extracellular lactate dehydrogenase (LDH) activity was measured from the cell culture media of 24-well plates using an enzyme kinetic photometric assay (Diagnosticum Ltd., Budapest, Hungary). Each well of a microplate was filled with 200 µL of the working reagent (56 mM phosphate buffer, pH = 7.5, 1.6 mM pyruvate, 240 M NADH + H^+^) and 10 µL of sample. A Multiskan GO 3.2 reader was used to detect the absorbance at 340 nm. The LDH activity was estimated by measuring the absorbance six times in one-minute increments while incubating the mixture at 37 °C and averaging the changes between the consecutive time points.

CXCLi2, IL-6, and Nrf2 concentrations were measured in the culture media, and protein carbonyl (PC) in the cell lysate samples of the 24-well plates with chicken-specific ELISA kits (MyBioSource, San Diego, CA, USA) following the manufacturer’s instructions. Absorbance values were obtained with a Multiskan GO 3.2 reader at 450 nm.

Total protein concentrations were measured from the cell lysate samples with the Pierce™ Bicinchoninic Acid (BCA) Protein Assay (Thermo Fisher Scientific, Waltham, MA, USA) as indicated by the manufacturer, with bovine serum albumin (BSA) as standard. 25 µL of sample was added to 200 µL of reagent mixture, and absorbance was measured after 30 min incubation at 37 °C at 562 nm with a Multiskan GO 3.2 reader. The values were used to standardize the PC values which were measured from the same cell lysate samples.

The protein concentration of IL-16, IFN-γ, IL-10, M-CSF and RANTES were measured from cell culture media using Luminex xMAP technology, performing Milliplex Chicken Cytokine/Chemokine Panel (Merck KGaA, Darmstadt, Germany) according to the instructions of the manufacturer. To summarize, all samples were thawed and examined in a blind-fashion and in duplicate. A 96-well plate was filled with 25 µL of each sample, standard, control, and assay buffer (provided with the kit). Each well received an additional 25 μL of five uniquely colored capture antibody-coated bead sets. Following overnight incubation, a biotinylated detection antibody combination and streptavidin phycoerythrin were applied to the plate following proper washing processes. After the last washing step, 150 mL drive fluid was added to the wells, the beads were resuspended for an additional 5 min on a plate shaker and read on the Luminex MAGPIX^®^ instrument. Data was collected using the Luminex xPonent 4.2 program. The Belysa^®^ Immunoassay Curve Fitting Software 1.2 (Merck KGaA, Darmstadt, Germany) software generated five-PL regression curves to draw the standard curves for all analytes using bead median fluorescence intensity (MFI) data.

To measure extracellular H_2_O_2_ levels in the culture media, the fluorometric Amplex Red technique (Thermo Fisher Scientific, Waltham, MA, USA) was utilized. After a 30-min incubation of 50 mL freshly prepared Amplex Red (100 M) and horseradish peroxidase (HRP) (0.2 U/mL) containing working solution with 50 mL culture medium at room temperature (21 °C), fluorescence (ex = 560 nm, em = 590 nm) was detected using a Victor X2 2030 fluorometer (Perkin Elmer, Waltham, MA, USA).

### Statistical analysis

All statistical analyses were performed in R v. 4.0.3 (R Core Team, 2020). Pairwise comparisons were performed using Wilcoxon signed-rank test^29^, as some of the treatment groups showed non-normal distribution based on Shappiro–Wilk tests^30^. We have considered a difference statistically significant if the p-value was less than 0.05. For visualization, the metabolic activity, the LDH activity, and the H_2_O_2_ values were converted to percentages. Letters above the boxes are used to indicate significant differences between groups. Different letters between two treatment groups mean that they were significantly different from each other.

## Results

All p-values for the pairwise comparisons performed by Wilcoxon sign-ranked test can be found in Supplementary information file 2. In this section, the significant changes that are considered biologically relevant are discussed.

**Figure 1:**
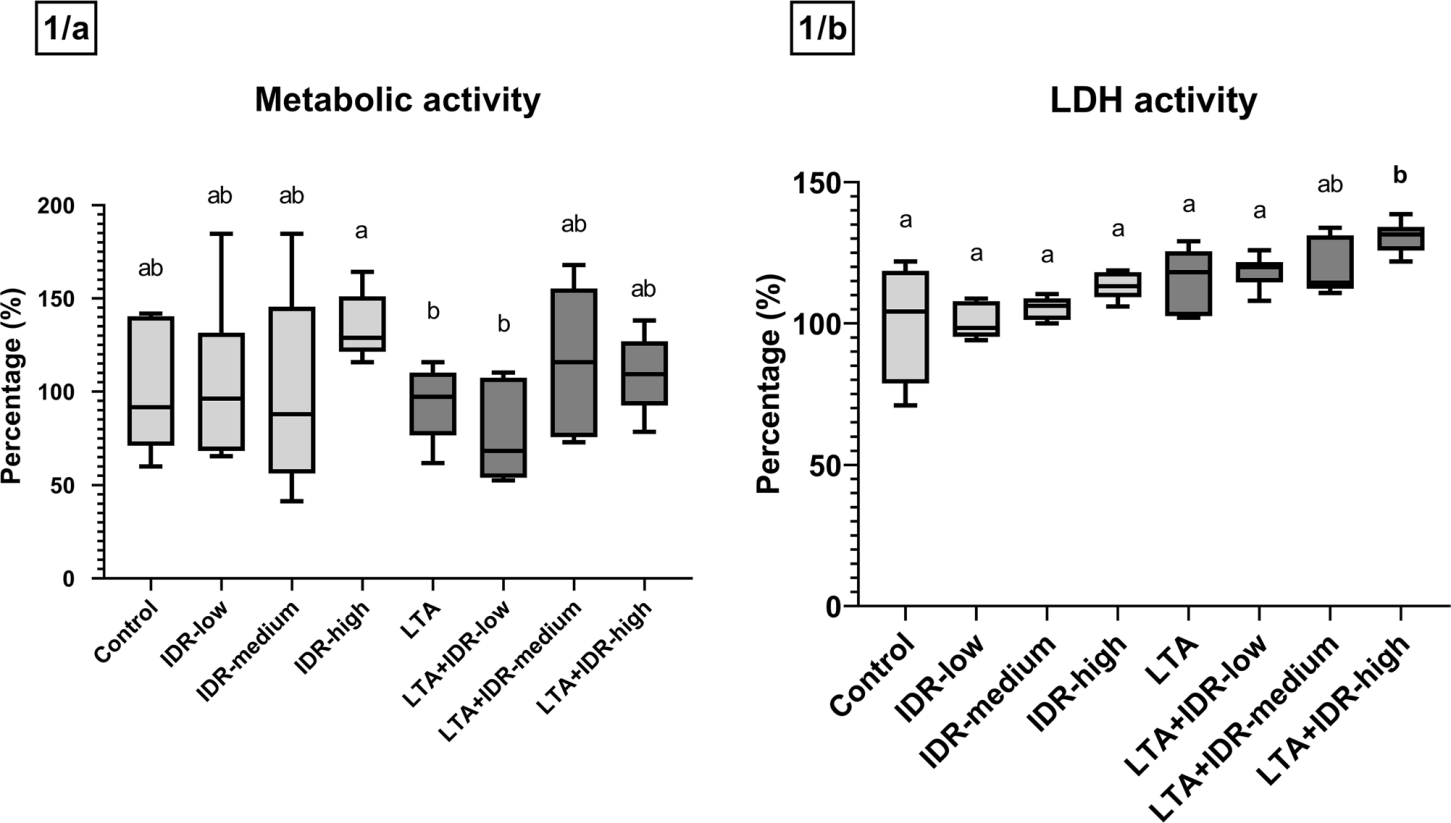
Metabolic activity (CCK-8 test) and LDH activity of the hepatocyte–non-parenchymal cell co-cultures from cell culture media in response to IDR-1002 and LTA treatment showed on boxplots. IDR-low = 10 µg/mL IDR-1002, IDR-medium = 30 µg/mL IDR-1002, IDR-high = 90 µg/mL IDR-1002, LTA = 50 µg/mL lipoteichoic acid from *Staphylococcus aureus* (n = 6/group). Cell cultures in Control group received none of the treatments. Results are displayed as percentage, where 100% is the mean value of control cultures. Wilcoxon sign-ranked tests were used to compare treatment groups to each other. Letters above the boxes are used to indicate significant differences between groups. Different letters between two treatment groups mean that they were significantly different from each other (p < 0.05). Significant results in which the groups receiving only IDR-1002 or LTA treatment differed from the Control group, or the groups receiving both LTA and IDR-1002 differed from the group receiving only LTA, are indicated in bold in the figure.

No significant changes in metabolic activity were observed either after IDR-1002, LTA, or combination treatments (Fig. 1a). IDR-1002 or LTA alone did not alter the extracellular LDH activity, however a significant increase was detected after treatment with LTA and 90 µg/mL concentration of IDR-1002 together compared to the LTA group (p = 0.015, Fig. 1b).

**Figure 2:**
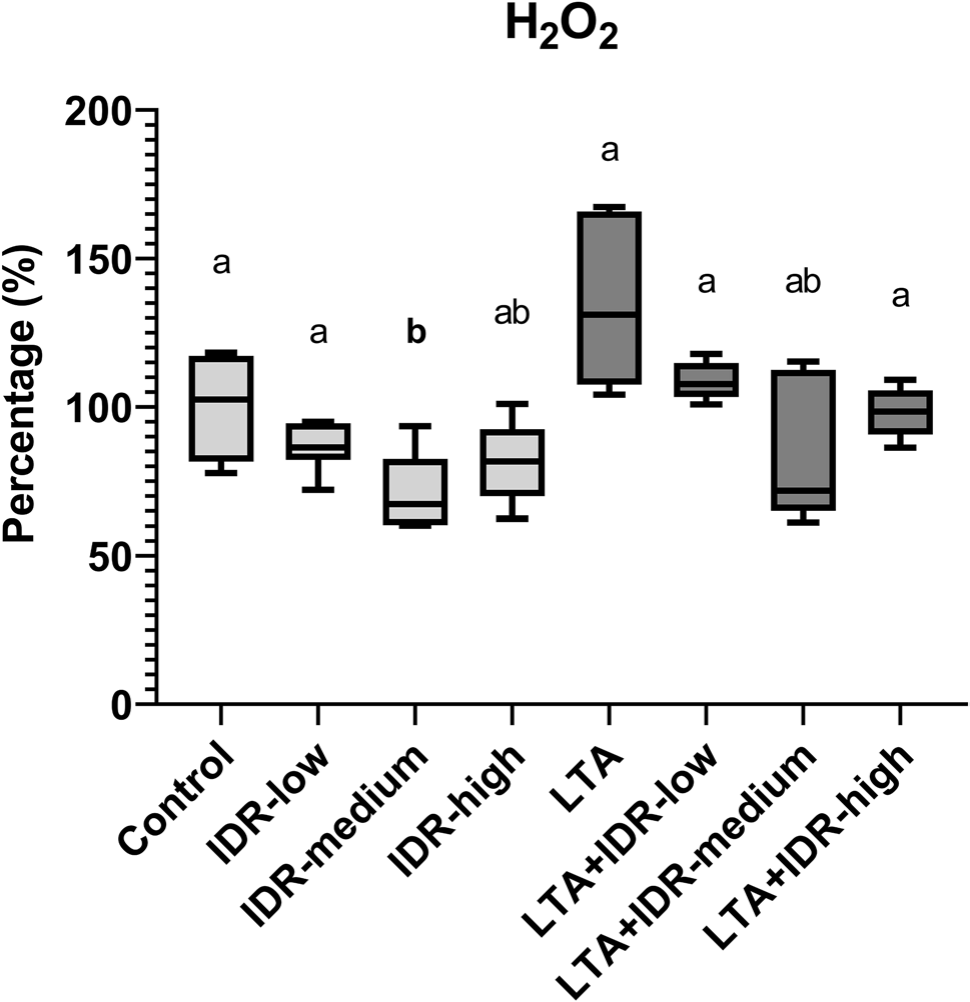
H_2_O_2_ levels of the hepatocyte–non-parenchymal cell co-cultures measured from cell culture media in response to IDR-1002 and LTA treatment showed on boxplots. IDR-low = 10 µg/mL IDR-1002, IDR-medium = 30 µg/mL IDR-1002, IDR-high = 90 µg/mL IDR-1002, LTA = 50 µg/mL lipoteichoic acid from *Staphylococcus* *aureus* (n = 6/group). Cell cultures in Control group received none of the treatments. Results are displayed as percentage, where 100% is the mean value of control cultures. Wilcoxon sign-ranked tests were used to compare treatment groups to each other. Letters above the boxes are used to indicate significant differences between groups. Different letters between two treatment groups mean that they were significantly different from each other (p < 0.05). Significant results in which the groups receiving only IDR-1002 or LTA treatment differed from the Control group, or the groups receiving both LTA and IDR-1002 differed from the group receiving only LTA, are indicated in bold in the figure.

**Figure 3:**
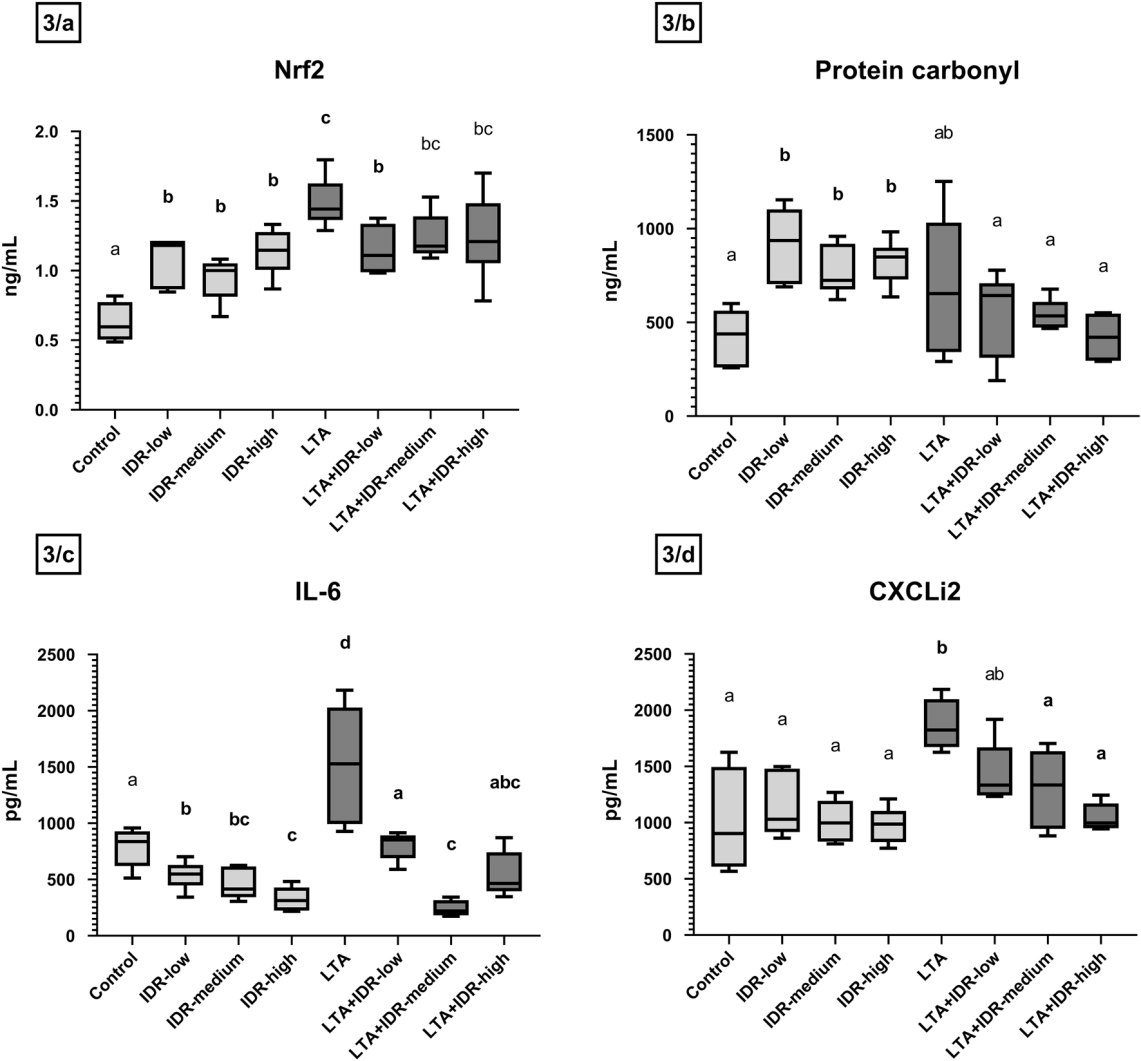
Nrf2, Protein carbonyl (PC), Interleukin-6 (IL-6) and CXCLi2 concentrations of the hepatocyte–non-parenchymal cell co-cultures measured with ELISA method from cell culture media (in the case of Nrf2, IL-6 and CXCLi2) and from cell lysate (in the case of PC) in response to IDR-1002 and LTA treatment showed on boxplots. IDR-low = 10 µg/mL IDR-1002, IDR-medium = 30 µg/mL IDR-1002, IDR-high = 90 µg/mL IDR-1002, LTA = 50 µg/mL lipoteichoic acid from *Staphylococcus* *aureus* (n = 6/group). Cell cultures in Control group received none of the treatments. Wilcoxon sign-ranked tests were used to compare treatment groups to each other. Letters above the boxes are used to indicate significant differences between groups. Different letters between two treatment groups mean that they were significantly different from each other (p < 0.05). Significant results in which the groups receiving only IDR-1002 or LTA treatment differed from the Control group, or the groups receiving both LTA and IDR-1002 differed from the group receiving only LTA, are indicated in bold in the figure.

Regarding the redox homeostasis, 30 µg/mL IDR-1002 decreased the H_2_O_2_ concentration (p = 0.03, Fig. 2), and all three concentrations of the peptide elevated the Nrf2 concentrations (p = 0.014, p = 0.032, p = 0.016, respectively, Fig. 3a). PC levels were also increased after treatment with all concentrations (p = 0.004 in all cases, Fig. 3b). LTA also elevated Nrf2 (p = 0.016, Fig. 3a) and in combination, 10 µg/mL IDR-1002 decreased it (p = 0.032, Fig. 3a). H_2_O_2_ levels were also decreased by the LTA + IDR-high treatment compared to LTA (p = 0.026, Fig. 2). Positive correlation was found between Nrf2 and PC in the IDR-low group as well, and a positive, but not significant one between Nrf2 and H_2_O_2_ in LTA + IDR-high (Supplementary Figs. 12, 13 in Supplementary Information File 3).

**Figure 4:**
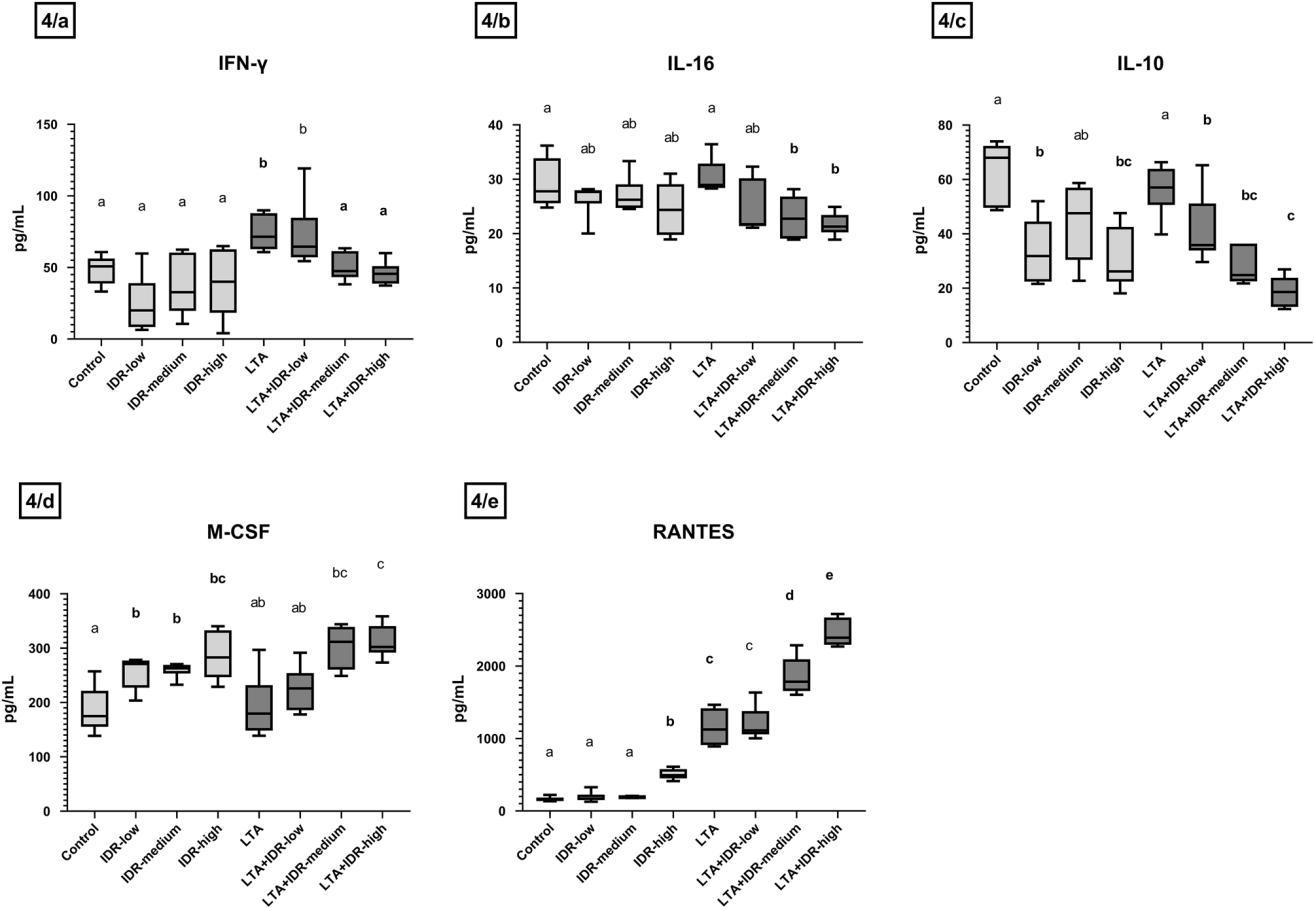
Interleukin-10 (IL-10), Interferon-γ (IFN-γ), IL-16, Macrophage Colony Stimulating Factor (M-CSF) and RANTES (CCL-5) concentrations of the hepatocyte–non-parenchymal cell co-cultures measured with Luminex method from cell culture media in response to IDR-1002 and LTA treatment showed on boxplots. IDR-low = 10 µg/mL IDR-1002, IDR-medium = 30 µg/mL IDR-1002, IDR-high = 90 µg/mL IDR-1002, LTA = 50 µg/mL lipoteichoic acid from *Staphylococcus* *aureus* (n = 6/group). Cell cultures in Control group received none of the treatments. Wilcoxon sign-ranked tests were used to compare treatment groups to each other. Letters above the boxes are used to indicate significant differences between groups. Different letters between two treatment groups mean that they were significantly different from each other (p < 0.05). Significant results in which the groups receiving only IDR-1002 or LTA treatment differed from the Control group, or the groups receiving both LTA and IDR-1002 differed from the group receiving only LTA, are indicated in bold in the figure.

LTA increased the concentrations of IL-6 (p = 0.016, Fig. 3c), CXCLi2 (p = 0.027, Fig. 3d), and IFN-γ (p = 0.009, Fig. 4a). These elevations were then decreased by IDR-1002, in the case of IL-6 by 10, 30 and 90 µg/mL (p = 0.004, p = 0.016, p = 0.004, respectively, Fig. 3c), CXCLi2 by 30 and 90 µg/mL (p = 0.019, p = 0.016, respectively, Fig. 3d), and IFN-γ by 30 and 90 µg/mL of IDR-1002 (p = 0.015, p = 0.002, respectively, Fig. 4a). After IDR-1002 treatment, IL-6 levels were decreased (IDR-low: p = 0.030, IDR-medium: p = 0.032, IDR-high: p = 0.004, Fig. 3c).

IL-16 and IL-10 concentrations were decreased compared to LTA after treatment with 30 and 90 µg/mL IDR-1002 (IL-16: p = 0.002, p = 0.005, respectively, Fig. 4b, IL-10: p = 0.002 in both cases, Fig. 4c). IL-10 concentrations were also decreased after IDR-1002 treatment (IDR-low: p = 0.009, IDR-high: p = 0.002, Fig. 4c).

M-CSF levels were elevated after treatment with 10, 30, 90 µg/mL of the peptide (p = 0.015, p = 0.004, p = 0.009, respectively, Fig. 4d), and RANTES levels after treatment with 90 µg/mL (p = 0.002, Fig. 4e). LTA also increased RANTES concentrations (p = 0.002), and IDR-1002 further elevated it compared to LTA (30 and 90 µg/mL: p = 0.002 in both cases, Fig. 4e). M-CSF levels were also increased in the combination groups compared to LTA (LTA + IDR-medium: p = 0.009, LTA + IDR-high: p = 0.004, Fig. 4d).

In addition, a strong positive correlation was found between RANTES and IFN-γ in the groups LTA + IDR-medium and -high, between RANTES and IL-16 in groups IDR-high and LTA + IDR-high, and interestingly, between RANTES and IL-10 in groups LTA + IDR-medium and -high. There were a negative correlation between M-CSF and CXCLi2 in the LTA + IDR-medium group, and a mentionable, but not significant positive correlation between M-CSF and IL-10 in the group LTA + IDR-medium. Details and plots are included as Supplementary information file 3 (Supplementary Figs. 2–11 in Supplementary Information File 3).

## Discussion

The main obstacle to the therapeutic use of natural HDPs is their adverse effects on the target organism, such as cytotoxicity, the stimulation of mast cell degranulation, and apoptosis^19,31–33^. The antimicrobial activity of these peptides can be explained by their interaction with membranes. It is generally accepted that naturally occurring, positively charged HDPs interact with negatively charged components of the cell membrane and then get inserted into the membrane in a parallel direction, thereby disrupting the continuity of the phospholipid bilayer. This allows the peptide to internalize and exert its intracellular effects^34^. However, high reactivity with the membrane, and thus potent antimicrobial activity may also be associated with toxic effects on the cells of the target organism. For example, LL-37 shows relatively high cytotoxicity not just towards bacterial, but eukaryotic cells as well^35–37^.

Previous research suggests that IDRs, while lacking direct antimicrobial function, do not have such adverse effects, and they need much higher concentrations in order to be cytotoxic^20,38,39^. In the present study, IDR-1002 was examined in concentrations of 10, 30, and 90 µg/mL, and did not alter the metabolic activity of the cells. There was a significant, moderate increase in extracellular LDH activity after treatment of the cells with both LTA and the highest concentration of the peptide together, suggesting some degree of membrane damage. Based on these results, we can conclude that none of the treatments caused a change in cell viability, although the high concentration of IDR-1002 treatment in the presence of an inflammatory stimulus can cause cell membrane damage. This indicates that, although the cell-damaging effects of the peptide are generally negligible, it would still be worth selecting the lowest effective concentration for therapeutic use.

IDRs gained attention in recent years through their ability to protect against bacterial infections without direct antimicrobial effect. They seem to modulate the host immune response by enhancing chemokine production and leukocyte recruitment, while suppressing harmful inflammation^14^. This study examined the effects of one peptide of the IDR family, IDR-1002, on the hepatic immune response of the chicken by measuring seven cytokines/chemokines (IL-6, CXCLi2, IFN-γ, IL-16, IL-10, M-CSF and RANTES).

One of the main aims of this experiment was to determine how the primary immune cells of the liver, Kupffer cells, respond to treatment with the peptide. For this purpose, cytokines that have a strong effect on macrophage differentiation were investigated. Firstly, the level of RANTES was measured in our experiment, and it was found that treatment with high concentrations of IDR-1002 (90 µg/mL) and LTA increased the concentration of this protein, and cell cultures treated with both IDR-1002 and LTA showed an even more pronounced concentration-dependent increase. RANTES, also known as CCL5 (C–C motif ligand 5), is a chemokine produced in the liver by hepatocytes, macrophage stellate cells, and endothelial cells. It stimulates leukocyte migration, modulates the production of several cytokines, and has a complex role in the regulation of inflammatory processes^40^. Other studies also shown similar results to ours, for example, treatment with IDR-1002 was found to be linked with an increase in RANTES levels in a murine infection model^23^, and IDR-1 was shown to stimulate signaling pathways contributing to the induction of RANTES^20^. In addition, IDR-1002 was found to activate the migration of monocytes towards RANTES, thereby stimulating bacterial elimination^41^.

RANTES has been shown to promote the conversion of macrophages to the M1 form (pro-inflammatory) and inhibit the M2 form (anti-inflammatory)^40^. In contrast, according to our findings, M-CSF levels were also increased after IDR-1002 treatment and were unchanged by LTA alone. M-CSF (also known as CSF-1) is produced by hepatocytes and Kupffer cells in the liver and plays an important role in the differentiation of Kupffer cells into M2-type macrophages and in the regulation of regenerative processes^7,42^. This seemingly contradictory result is in line with research that found, based on gene expression studies, that IDR-1018 promoted a phenotypic transition between the two forms^21^, while LL-37 stimulated M1 macrophage formation^43^. Both types of macrophages play an important role in inflammatory processes. M2 macrophages are able to perform active phagocytosis and produce high levels of chemotactic factors and IL-10, and are therefore usually referred to as anti-inflammatory cells^44^. M1 macrophages, in contrast, produce pro-inflammatory cytokines and have anti-tumour immune promoting activity, but can also play a role in tissue damage^45^.

Although the levels of certain pro-inflammatory cytokines decreasing following IDR-1002 treatment (IL-6, IL-16) would suggest that macrophages were preferentially recruited in the anti-inflammatory M2 form promoted by M-CSF, we found that IL-10 levels also decreased following treatment with the peptide (and were not affected by LTA). These findings indicate the predominance of a process driven by RANTES. This is further confirmed by the strong positive correlations which were found between RANTES and IFN-γ, between RANTES and IL-16, and interestingly, between RANTES and IL-10 (see Supplementary Figs. 4–6 in Supplementary information file 3). In addition, there was a negative correlation between M-CSF and CXCLi2, and a mentionable, but not significant, positive correlation between M-CSF and IL-10. This supports the idea that macrophages stimulated by IDR-1002 remain in a transition state between the two phenotypes.

As mentioned above, the decrease of IL-6 concentration was observed after treatment with IDR-1002. Notwithstanding that IDR-1002 has been shown to increase the production of these chemokines in various studies^23–25^, the effect of the peptide on different immune cells is not fully understood, and to the best of our knowledge, no research has been published on its action on hepatocytes and Kupffer cells. In the work of Huante-Mendoza et al.^22^, it was found that the translocation of NF-κB into the nucleus and the CREB (cAMP-response element binding protein)-CBP (CREB binding protein) complex formation were inhibited by the peptide after LPS treatment, suggesting that the induction of pro- and anti-inflammatory chemokines (such as IL-6, IL-8 or IL-10) was obstructed. Furthermore, IDR-1002 suppressed the inflammation induced by *Pseudomonas aeruginosa* infection in mouse lungs, and did not enhance the production of the tested cytokines, including IL-6 and TNF-α^24^.

IFN-γ is known to activate macrophages and promote pro-inflammatory cytokine production-, while suppressing the production of anti-inflammatory cytokines^46^. The production of IFN- γ by Kupffer cells can also lead to acute liver damage, or worsen existing chronic hepatic failure^47,48^. Following LTA treatment, IFN-γ levels were elevated, indicating the pro-inflammatory effect of the applied endotoxin. This effect was reduced by IDR-1002 treatment at all three concentrations, suggesting that the peptide was successful in alleviating the inflammatory response.

IDR-1002 did not significantly affect the levels of CXCLi2 (corresponding to IL-8 in chickens) when solely applied in our experiments. The concentration of this cytokine was, however, increased by LTA treatment, and reduced by simultaneous application of LTA and 30 and 90 µg/mL concentrations of the peptide. Similar results were shown in a previous study where IDR-1 successfully reduced LPS-induced IL-8 activation^20^, and also where IDR-1002 dampened IL-1β-induced IL-8 production^49^. Similarly to these results, LTA-induced IL-6 levels were also diminished by IDR-1002, but in this case, treatment with the peptide alone also decreased the level of this interleukin. Previous studies showed that IDR-1002 was able to reduce IL-6 production in a *Pseudomonas aeruginosa* lung infection model^24,25^, and in a *Staphylococcus aureus* treated RAW 264.7 macrophage cell line^27^.

In the present study, IL-16 levels were decreased in cells co-treated with LTA and IDR-1002. IL-16 is a chemokine that stimulates the migration of CD14^+^ immune cells, may cause tissue damage through pro-inflammatory processes, and plays an important role in mediating liver cell apoptosis. Its reduced levels may have several advantages in the event of an inflammatory response^50,51^.

Phagocytosis of various pathogenic microorganisms results in the formation of lysosomes, which contain high amounts of reactive oxygen species (ROS) and reactive nitrogen species (RNS)^52^. The associated oxidative stress helps to eliminate pathogens, but their excessive production can cause tissue damage, and they have also been shown to play a role in acute liver injury^53–55^. As it did with the pro-inflammatory cytokines IL-6 and IFN-γ, IDR-1002 also reduced the level of H_2_O_2_ in the medium (at a concentration of 30 µg/mL), which may also contribute to the reduction of cell damage.

**Figure 5:**
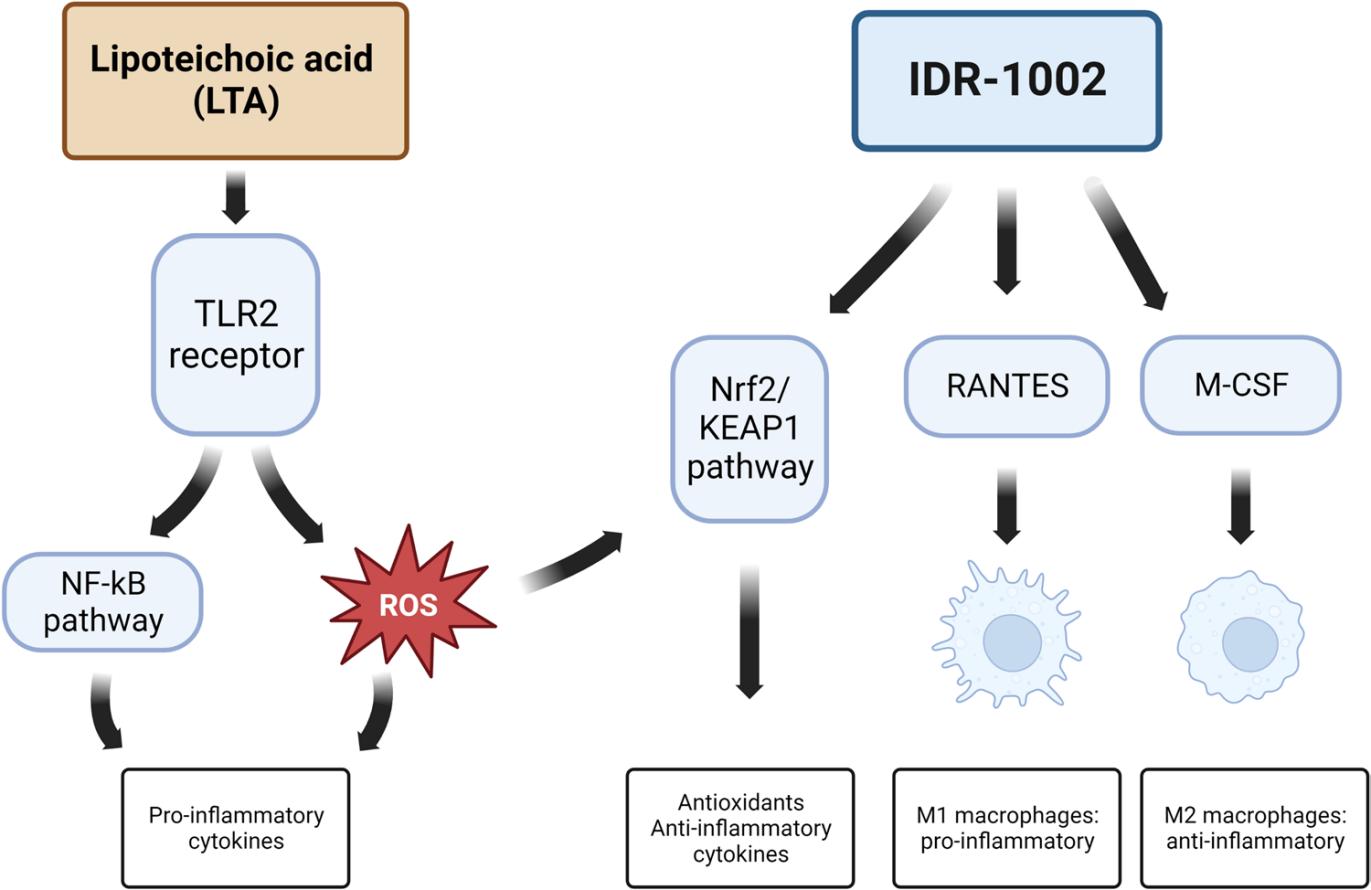
The effects of lipoteichoic acid (LTA) and Innate Defense Regulator-1002 (IDR-1002) on the inflammatory and antioxidant pathways in the liver. *TLR2* Toll like receptor 2, *NF-κB* Nuclear factor kappa light chain enhancer of activated B cells, *ROS* Reactive oxygen species, *Nrf2/KEAP1* Nuclear factor erythroid 2-related factor 2/Kelch-like ECH-associated protein 1, *RANTES* Regulated on Activation, Normal T cell Expressed and Secreted, *M-CSF* Macrophage colony stimulating factor. This figure was created with BioRender.com.

The IDR-1002-driven decrease in ROS level could be explained by the elevated levels of the transcription factor Nrf2 in IDR-1002 treated cells. The signaling pathway regulated by Nrf2/KEAP1 (nuclear factor erythroid 2-related factor 2/Kelch-like ECH-associated protein 1) plays an important role in protecting cells from oxidative stress. Nrf2 is degraded in the proteosomes in reaction with KEAP, but in the presence of ROS, RNS or certain protective molecules (e.g. isothiocyanates, organosulfur compounds, polyphenols), the cysteine residues of KEAP1 are oxidized, leading to the dissociation of Nrf2 and its ability to enter the nucleus. Once the Nrf2 is there, it can bind to various AREs (antioxidant responsive elements), leading to the activation of numerous antioxidant systems (e.g. superoxide dismutase, glutathione peroxidase)^54,56^. In addition, LTA also increased Nrf2 levels, which may have been a compensatory response due to the role Nrf2 plays in the regulation of innate immunity by providing protection against excessive inflammatory processes. It has been shown that ROS levels elevated by LTA or LPS activated the translocation of Nrf2, triggering the cellular defense system^57,58^. The interlinking between different antioxidant and inflammatory signaling pathways activated by LTA and IDR-1002 are shown in Fig. 5.

Interestingly, carbonylated protein levels were elevated by all three concentrations of IDR-1002. This outcome could be connected to the fact that some HDPs/AMPs (including IDR-1002) have been found to have potent antibiofilm effects^59–61^. As biofilms contain large amount of proteins^62^, it can be beneficial for the peptide to be able to carbonylate them. For example, the AMP KWI18 has been found to inhibit biofilm formation while inducing lipid and protein oxidation^63^. In our case, PC formation was not linked with ROS production and did not cause any damage to the cells. Based on these results, the role of IDR-1002 in oxidative stress may be worth further investigation by including other parameters.

In conclusion, it can be stated that IDR-1002 has a complex effect on regulating the immune status of hepatic cells in chickens under in vitro conditions. The peptide was shown to reduce the LTA-induced pro-inflammatory response (as seen for IFN-γ, IL-6, CXCLi2, and IL-16), suggesting that this HDP may be a promising candidate for attenuating pathological inflammatory processes. Moreover, the measured parameters suggested that macrophage activity was modulated to an intermediate state between anti- and pro-inflammatory forms, as IDR-1002 reduced both the concentration of pro-inflammatory cytokines (IFN-γ, IL-6) and the anti-inflammatory IL-10. This effect can be well explained by the increase in the level of RANTES (redirecting macrophages towards the pro-inflammatory M1 type) and M-CSF (stimulating differentiation towards the anti-inflammatory M2 type). In addition, we can conclude that IDR-1002 also affects the oxidative status of cells by reducing the levels of extracellular H_2_O_2_, presumably due to its inductive effect on the Nrf2 signaling pathway. The peptide was also able to attenuate LTA-induced H_2_O_2_ production accompanied by Nrf2 induction. Thus, it can be stated that IDR-1002 has a highly complex effect on the cellular immune response and, although further extensive studies are undoubtedly needed to elucidate its exact mechanism, this HDP may be a promising candidate for the treatment of pathologies associated with inflammation of bacterial origin. These results are particularly relevant given the limited research on the application of HDPs in the field of livestock science, especially in poultry farming. Furthermore, modern animal husbandry plays an important part in the extensive spread of antimicrobial resistance, which is one of the most critical concerns for our future. As a result, research into alternative agents such as HDPs is of key importance to identify the most suitable and practically applicable drug candidates for the future.

### Supplementary Information

Supplementary Information 1.

Supplementary Information 2.

Supplementary Figures.

Supplementary Legends.

## Data Availability

All data generated or analyzed during this study are included in this published article (and its Supplementary Information files).
