# Supplementary material for: Modulation of the immune response by the host defense peptide IDR-1002 in chicken hepatic cell culture
Source: Sci Rep. 2023 Sep 4;13:14530. doi: 10.1038/s41598-023-41707-z (PMC10477227; doi:10.1038/s41598-023-41707-z)
Supplement: Supplementary file 3 — Supplementary Figures. [file 41598_2023_41707_MOESM3_ESM.pdf]

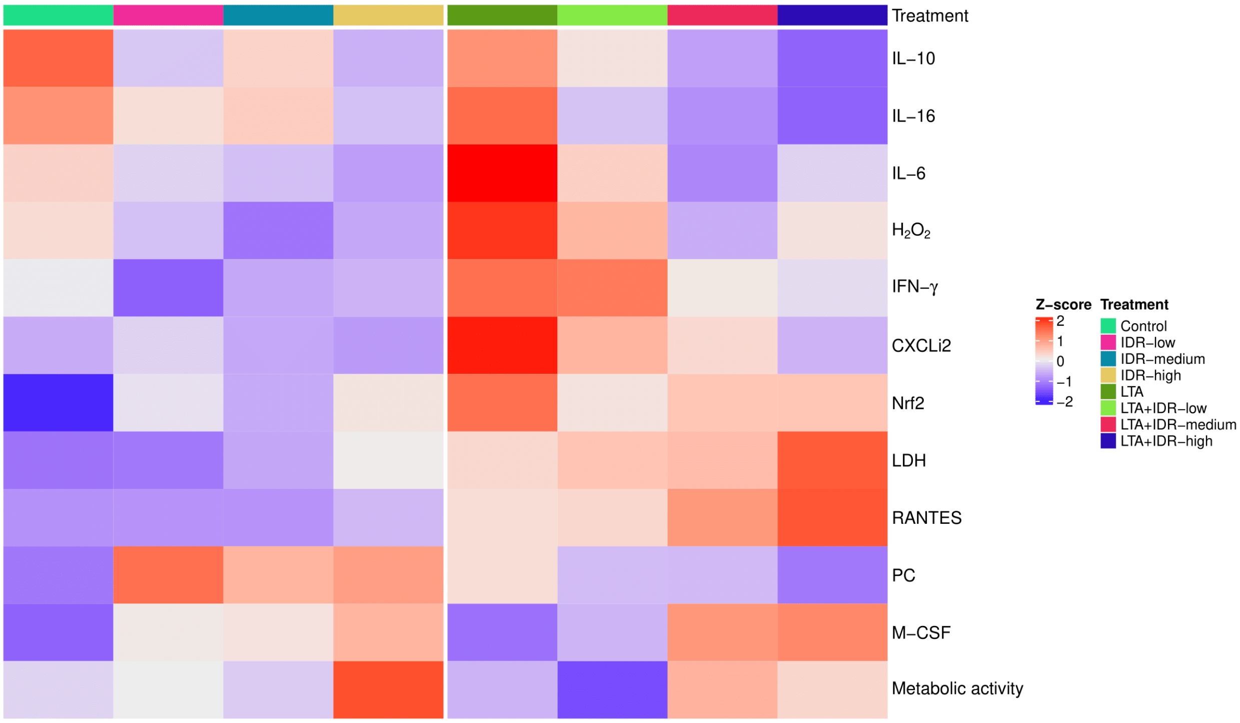

<u>Supplementary Figure 1:</u> Heatmap showing the average of the outcome of each parameter measured in the present study.

Mean of measurement results (presented in the rows) are Z-score transformed for comparability. Different colors indicate treatment groups (presented in the columns). Rows were clustered for better readability of the plot (dendogram not shown). IDR-low = 10 µg/mL IDR-1002, IDR-medium = 30 µg/mL IDR-1002, IDR-high = 90 µg/mL IDR-1002, LTA = 50 µg/mL lipotheichoic acid from *Staphylococcus aureus* (n = 6/group). Cell cultures in Control group received none of the treatments.

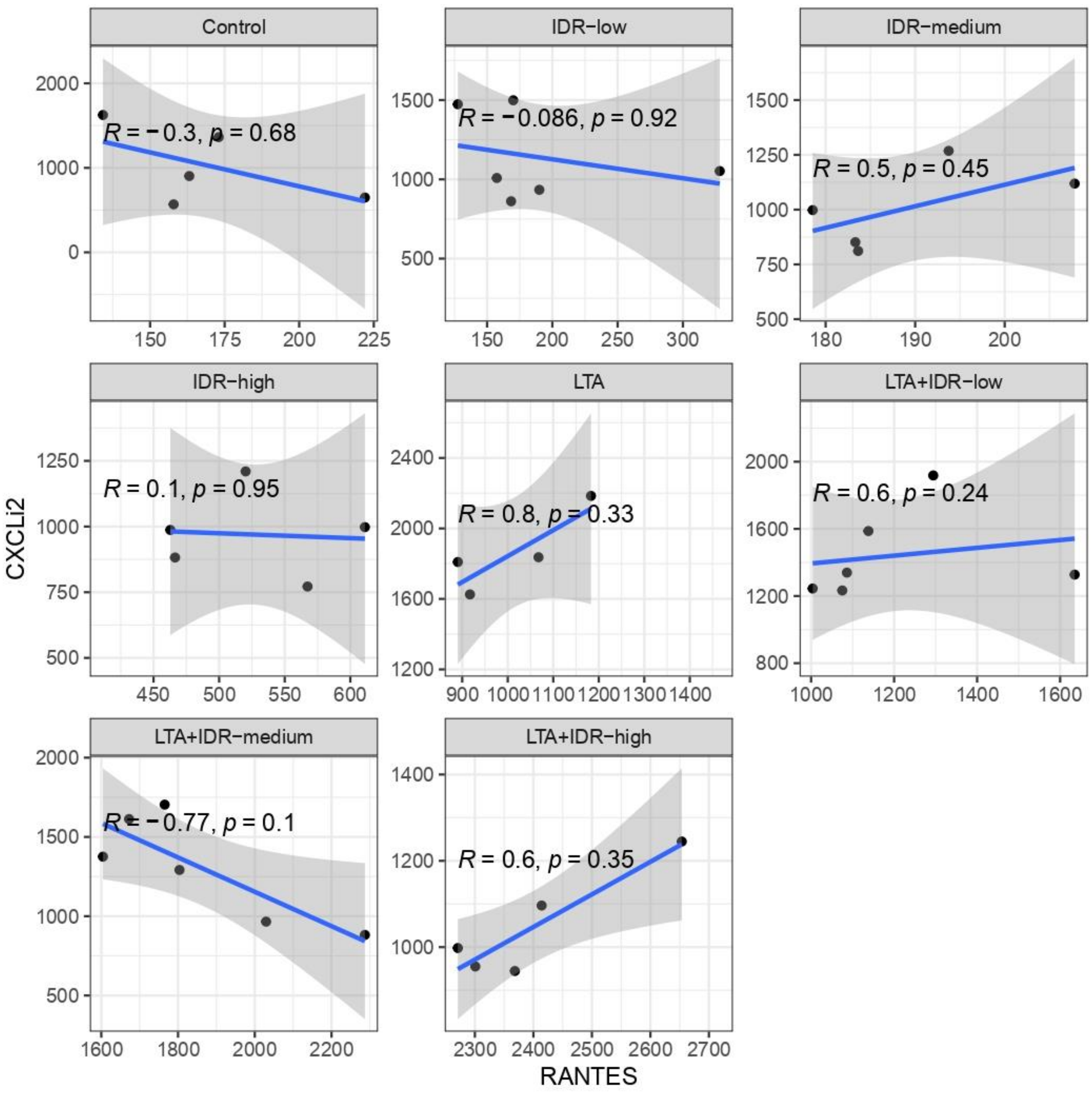

<u>Supplementary Figure 2:</u> **Scatterplot showing the association between RANTES and CXCLi2 values for each treatment group.**

Linear regression lines (with 95% confidence band) and Spearman correlation coefficients with p-values are displayed on each subplot for every treatment group. IDR-low = 10 µg/mL IDR-1002, IDR-medium = 30 µg/mL IDR-1002, IDR-high = 90 µg/mL IDR-1002, LTA = 50 µg/mL lipotheichoic acid from Staphylococcus aureus (n = 6/group). Cell cultures in Control group received none of the treatments.

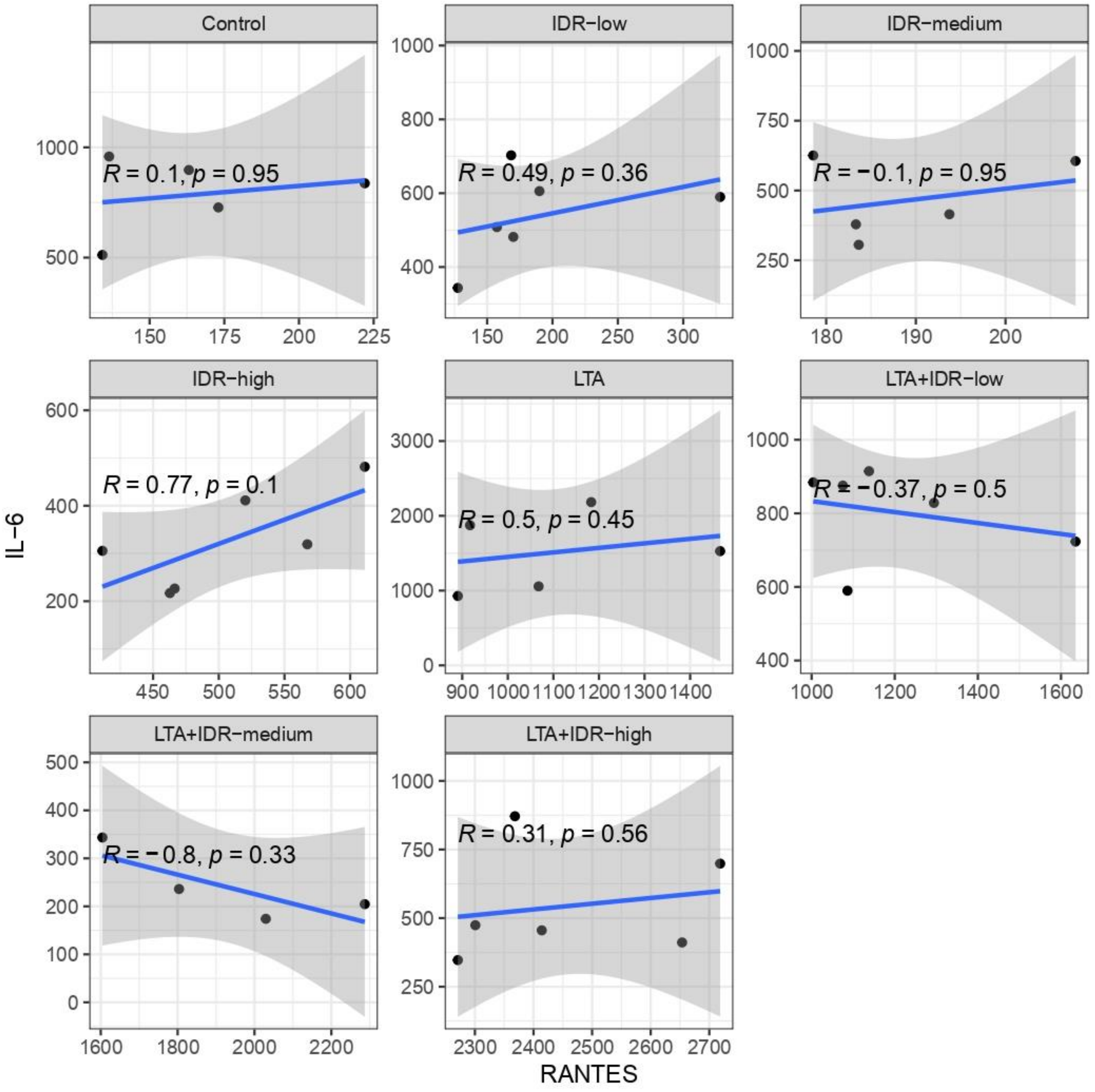

<u>Supplementary Figure 3:</u> Scatterplot showing the association between RANTES and Interleukin (IL)-6 values for each treatment group.

Linear regression lines (with 95% confidence band) and Spearman correlation coefficients with p-values are displayed on each subplot for every treatment group. IDR-low = 10 µg/mL IDR-1002, IDR-medium = 30 µg/mL IDR-1002, IDR-high = 90 µg/mL IDR-1002, LTA = 50 µg/mL lipotheichoic acid from Staphylococcus aureus (n = 6/group). Cell cultures in Control group received none of the treatments.

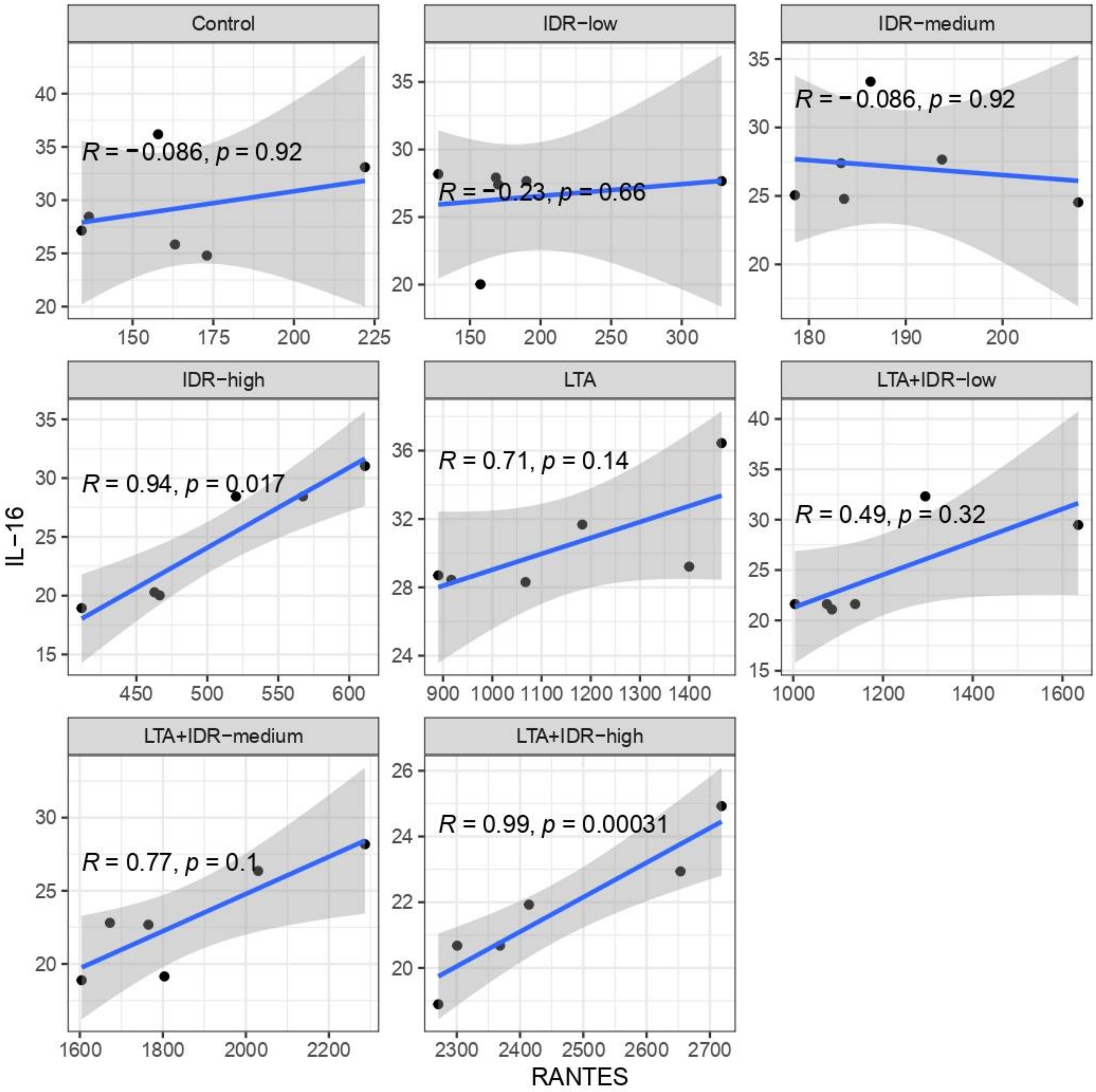

<u>Supplementary Figure 4:</u> Scatterplot showing the association between RANTES and Interleukin (IL)-16 values for each treatment group.

Linear regression lines (with 95% confidence band) and Spearman correlation coefficients with p-values are displayed on each subplot for every treatment group. IDR-low = 10 µg/mL IDR-1002, IDR-medium = 30 µg/mL IDR-1002, IDR-high = 90 µg/mL IDR-1002, LTA = 50 µg/mL lipotheichoic acid from Staphylococcus aureus (n = 6/group). Cell cultures in Control group received none of the treatments.

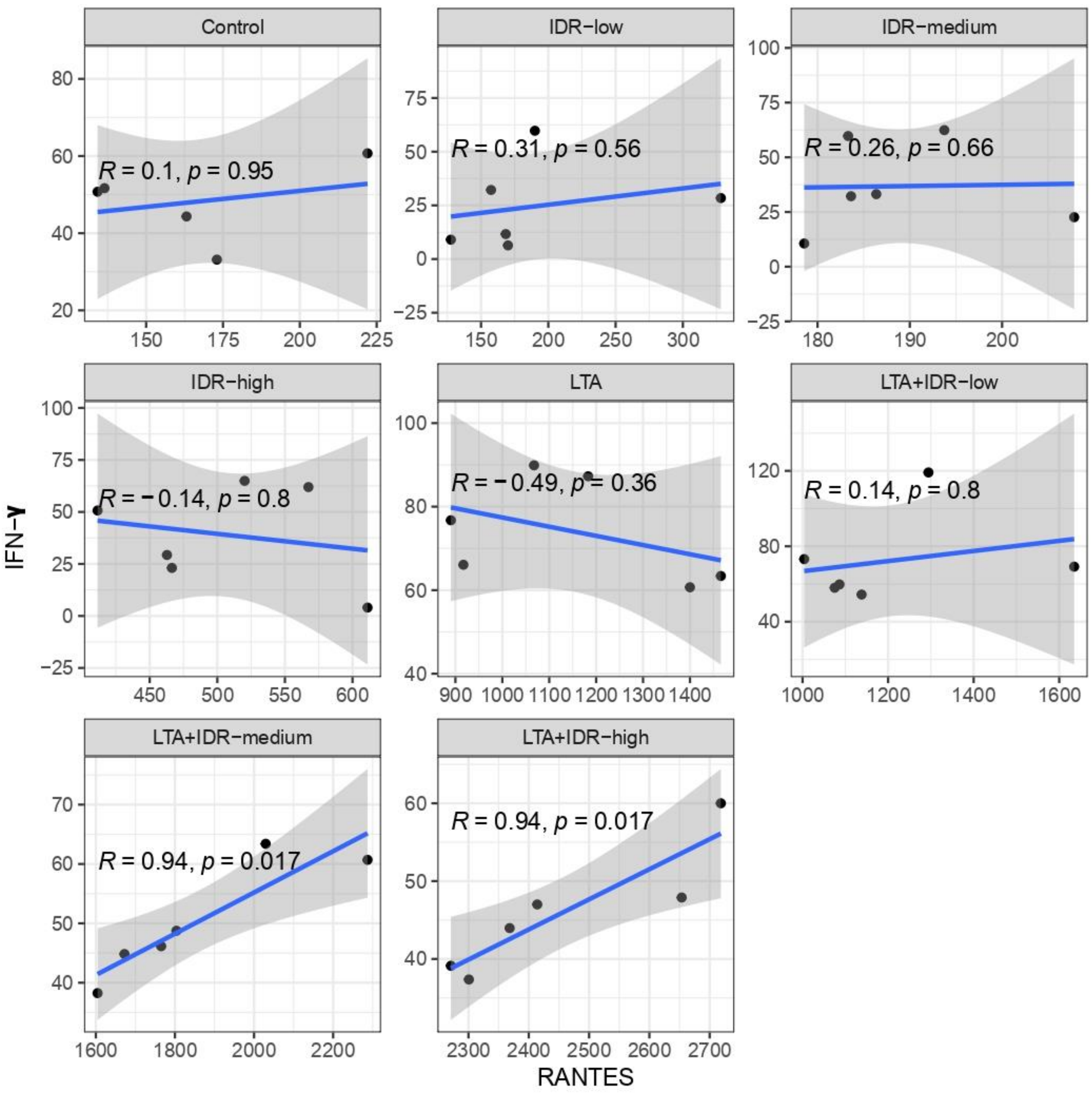

<u>Supplementary Figure 5:</u> Scatterplot showing the association between RANTES and Interferon (IFN)-γ values for each treatment group.

Linear regression lines (with 95% confidence band) and Spearman correlation coefficients with p-values are displayed on each subplot for every treatment group. IDR-low = 10 µg/mL IDR-1002, IDR-medium = 30 µg/mL IDR-1002, IDR-high = 90 µg/mL IDR-1002, LTA = 50 µg/mL lipotheichoic acid from Staphylococcus aureus (n = 6/group). Cell cultures in Control group received none of the treatments.

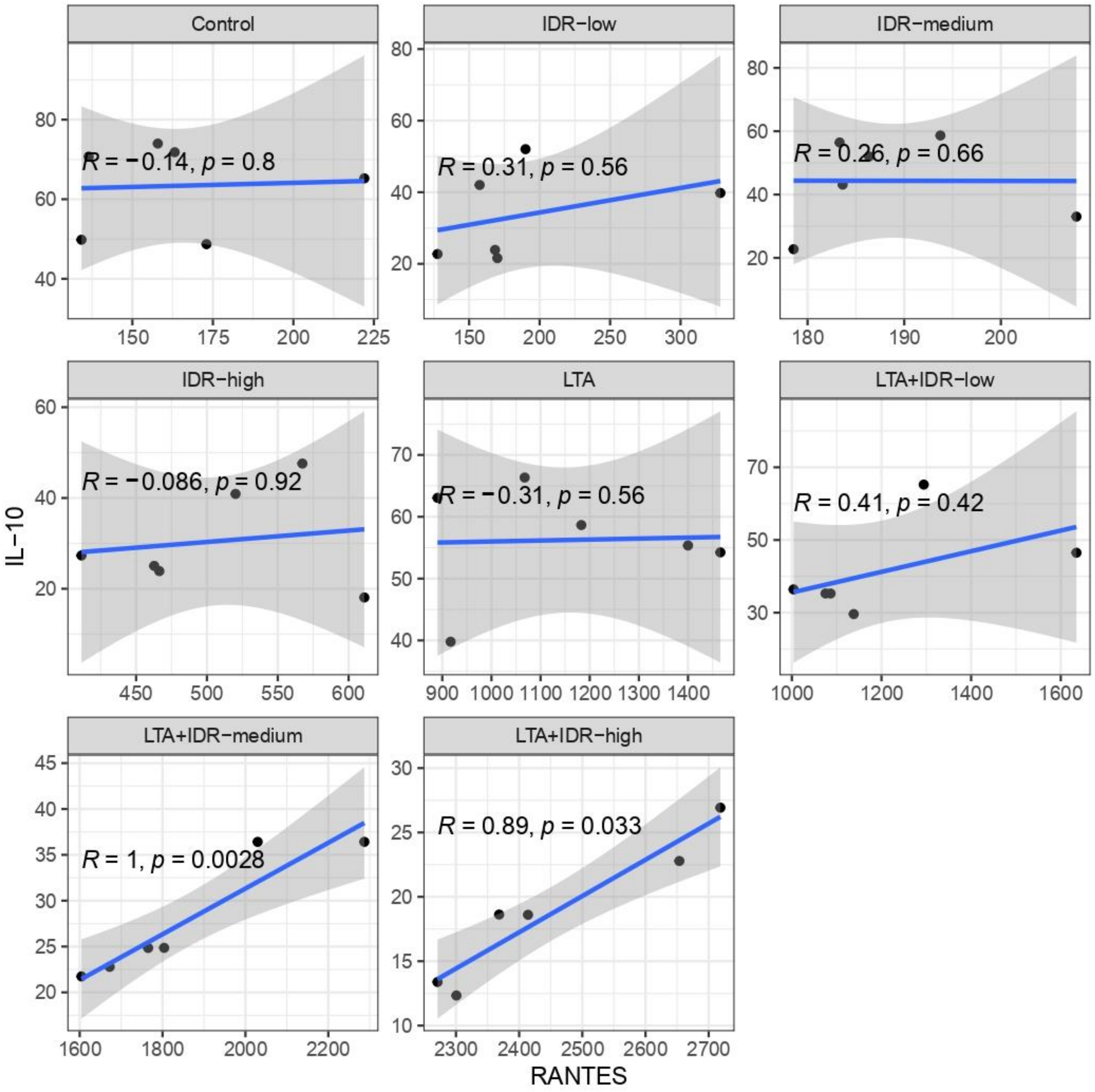

<u>Supplementary Figure 6:</u> Scatterplot showing the association between RANTES and Interleukin (IL)-10 values for each treatment group.

Linear regression lines (with 95% confidence band) and Spearman correlation coefficients with p-values are displayed on each subplot for every treatment group. IDR-low = 10 µg/mL IDR-1002, IDR-medium = 30 µg/mL IDR-1002, IDR-high = 90 µg/mL IDR-1002, LTA = 50 µg/mL lipotheichoic acid from Staphylococcus aureus (n = 6/group). Cell cultures in Control group received none of the treatments.

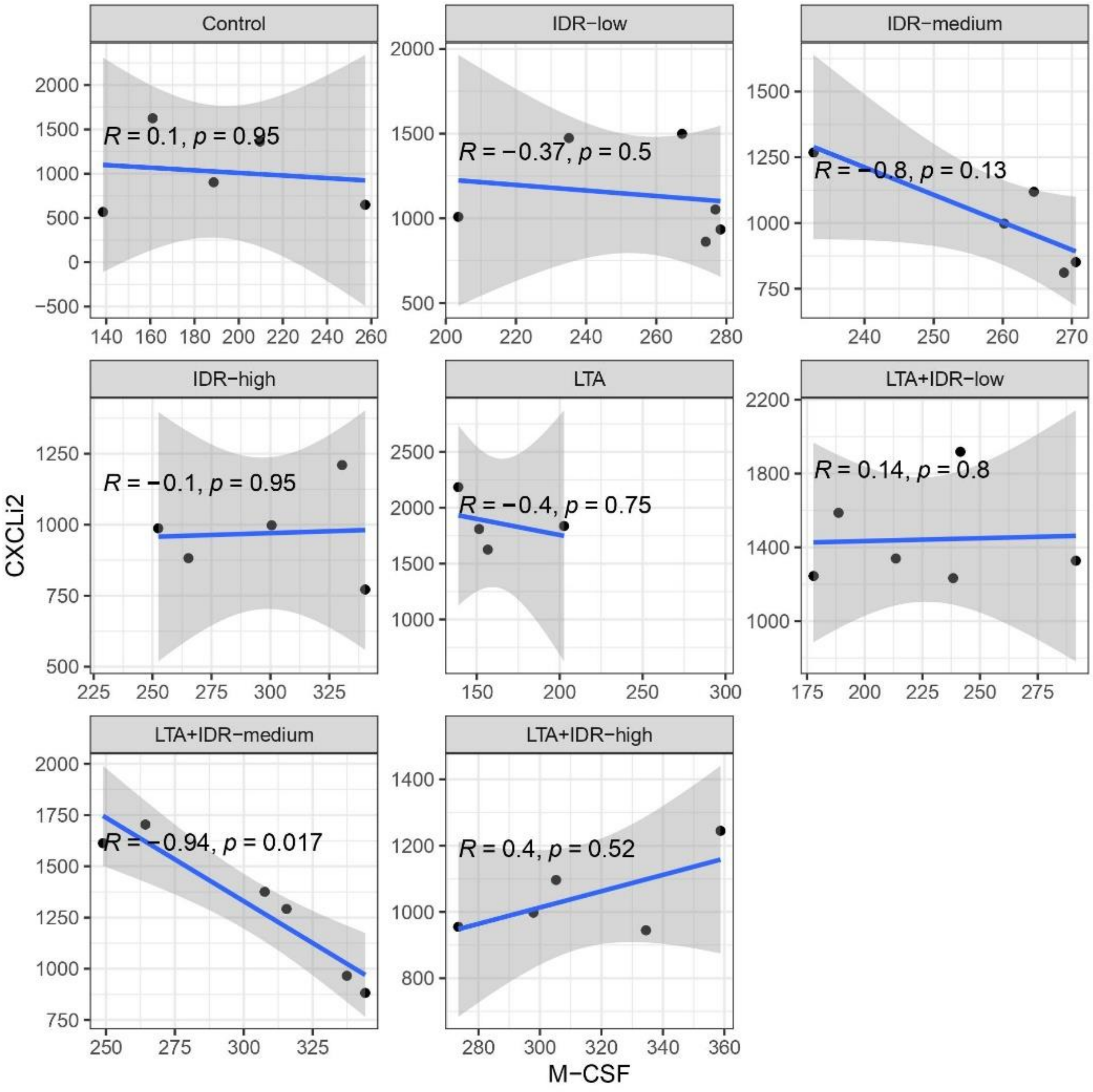

<u>Supplementary Figure 7:</u> Scatterplot showing the association between M-CSF and CXCLi2 values for each treatment group. Linear regression lines (with 95% confidence band) and Spearman correlation coefficients with p-values are displayed on each subplot for every treatment group. IDR-low = 10 µg/mL IDR-1002, IDR-medium = 30 µg/mL IDR-1002, IDR-high = 90 µg/mL IDR-1002, LTA = 50 µg/mL lipotheichoic acid from Staphylococcusaureus (n = 6/group). Cell cultures in Control group received none of the treatments.

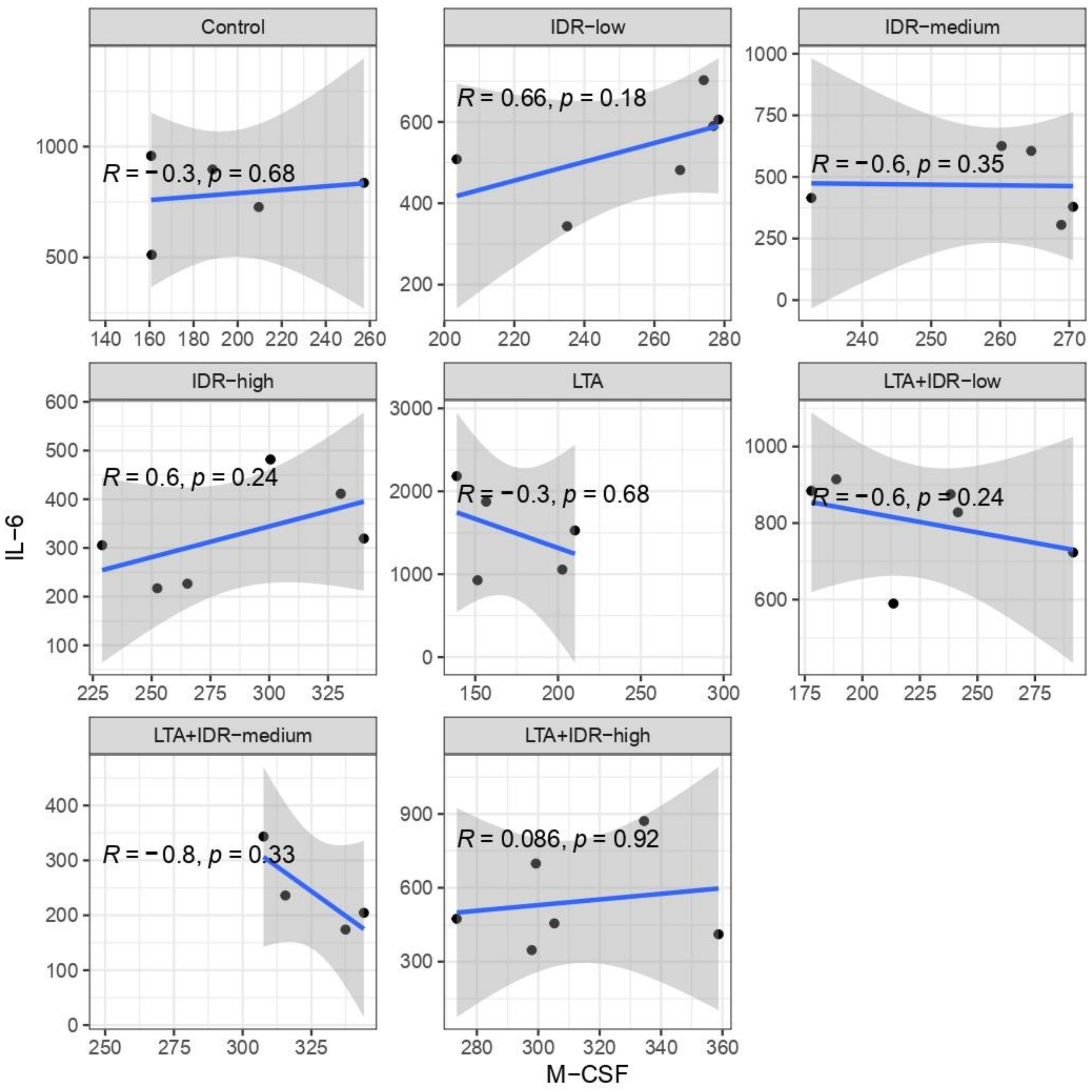

<u>Supplementary Figure 8:</u> Scatterplot showing the association between M-CSF and Interleukin (IL)-6 values for each treatment group.

Linear regression lines (with 95% confidence band) and Spearman correlation coefficients with p-values are displayed on each subplot for every treatment group. IDR-low = 10 µg/mL IDR-1002, IDR-medium = 30 µg/mL IDR-1002, IDR-high = 90 µg/mL IDR-1002, LTA = 50 µg/mL lipotheichoic acid from Staphylococcus aureus (n = 6/group). Cell cultures in Control group received none of the treatments.

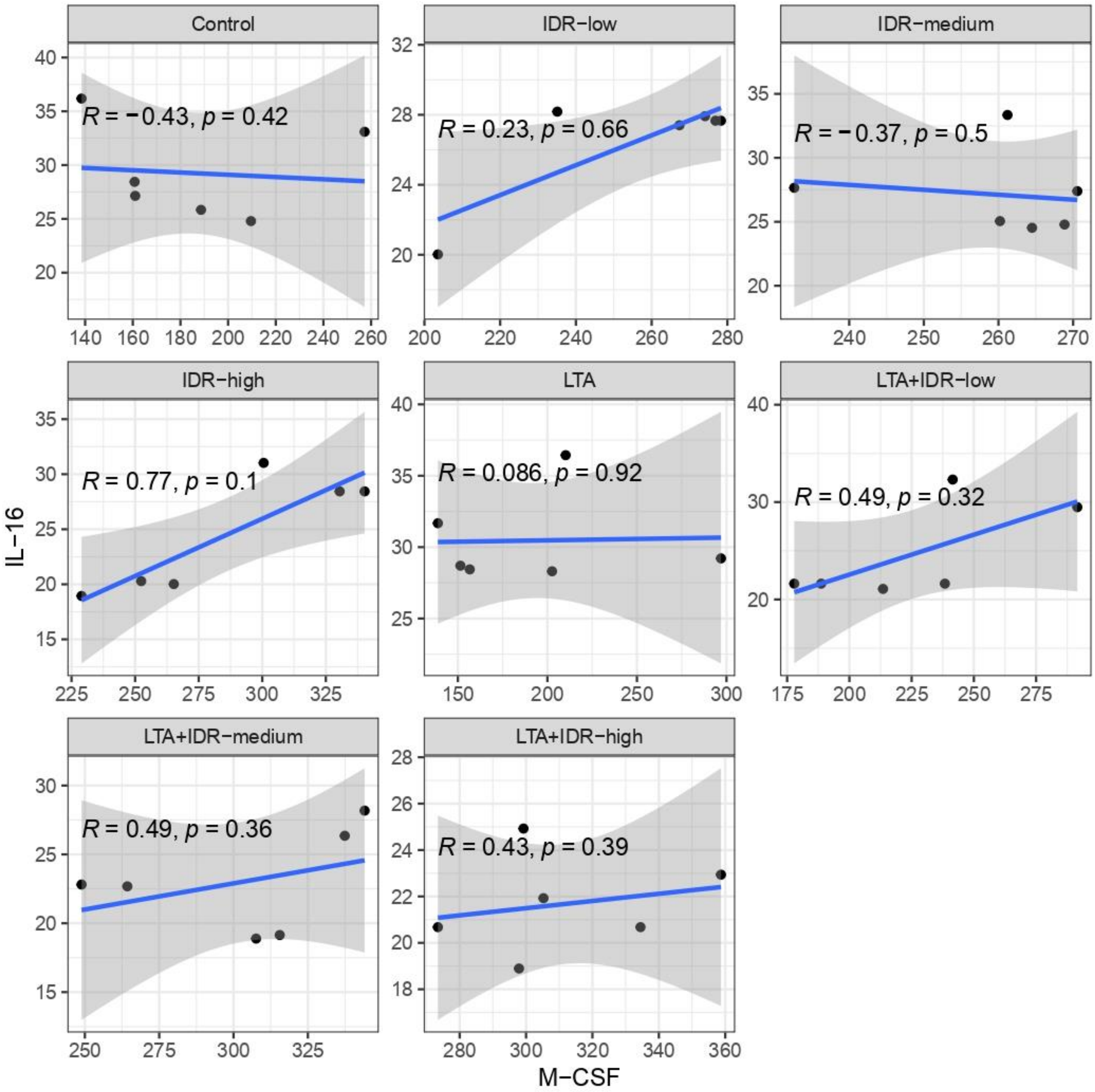

<u>Supplementary Figure 9:</u> Scatterplot showing the association between M-CSF and Interleukin (IL)-16 values for each treatment group.

Linear regression lines (with 95% confidence band) and Spearman correlation coefficients with p-values are displayed on each subplot for every treatment group. IDR-low = 10 µg/mL IDR-1002, IDR-medium = 30 µg/mL IDR-1002, IDR-high = 90 µg/mL IDR-1002, LTA = 50 µg/mL lipotheichoic acid from Staphylococcus aureus (n = 6/group). Cell cultures in Control group received none of the treatments.

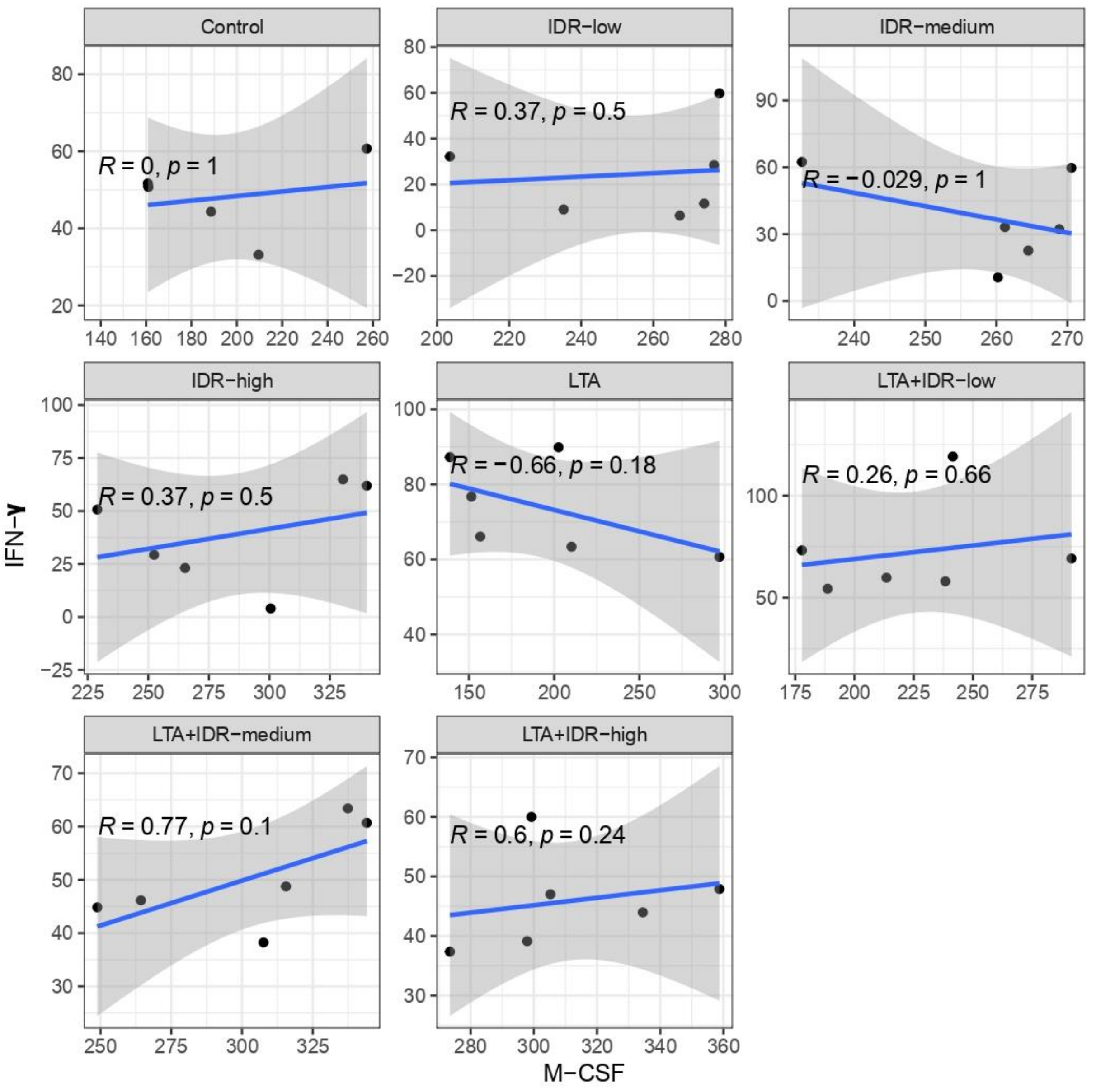

<u>Supplementary Figure 10:</u> Scatterplot showing the association between M-CSF and Interferon (IFN)-γ values for each treatment group.

Linear regression lines (with 95% confidence band) and Spearman correlation coefficients with p-values are displayed on each subplot for every treatment group. IDR-low = 10 μg/mL IDR-1002, IDR-medium = 30 μg/mL IDR-1002, IDR-high = 90 μg/mL IDR-1002, LTA = 50 μg/mL lipotheichoic acid from Staphylococcus aureus (n = 6/group). Cell cultures in Control group received none of the treatments.

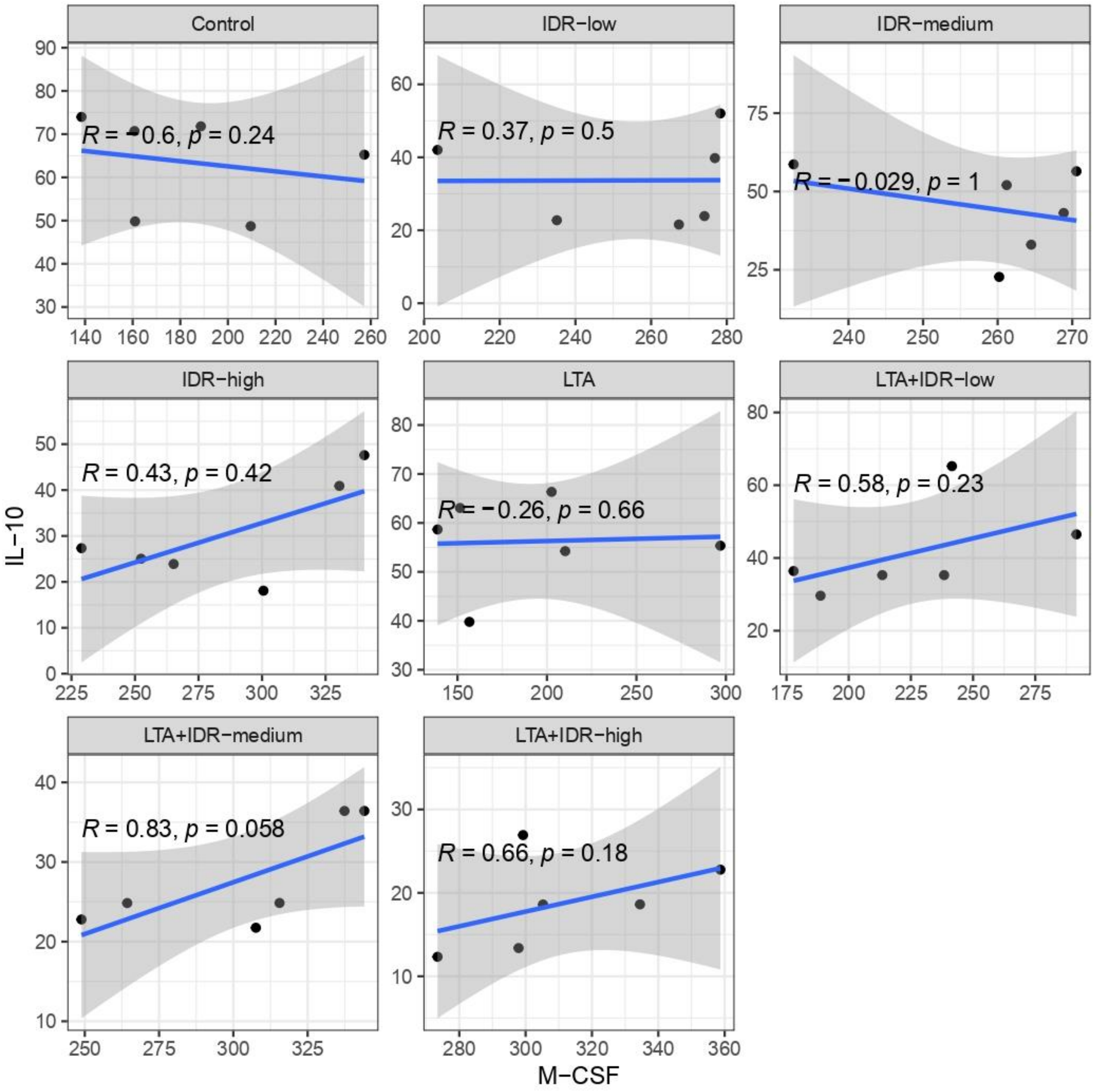

Supplementary Figure 11: Scatterplot showing the association between M-CSF and Interleukin (IL)-10 values for each treatment group.

Linear regression lines (with 95% confidence band) and Spearman correlation coefficients with p-values are displayed on each subplot for every treatment group. IDR-low = 10 µg/mL IDR-1002, IDR-medium = 30 µg/mL IDR-1002, IDR-high = 90 µg/mL IDR-1002, LTA = 50 µg/mL lipotheichoic acid from Staphylococcus aureus (n = 6/group). Cell cultures in Control group received none of the treatments.

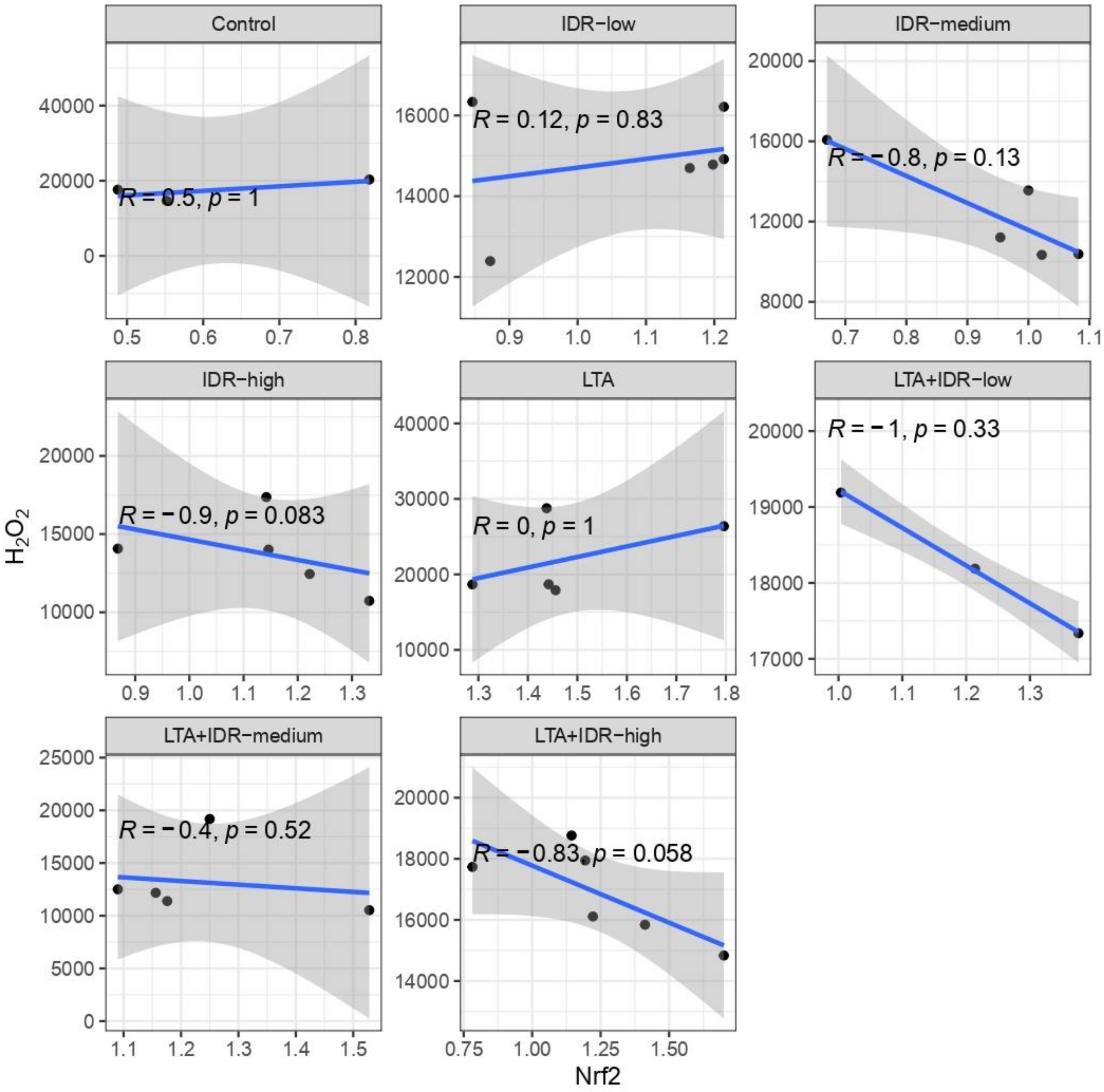

<u>Supplementary Figure 12:</u> Scatterplot showing the association between Nrf2 and H_2O_2 values for each treatment group.

Linear regression lines (with 95% confidence band) and Spearman correlation coefficients with p-values are displayed on each subplot for every treatment group. IDR-low = 10 µg/mL IDR-1002, IDR-medium = 30 µg/mL IDR-1002, IDR-high = 90 µg/mL IDR-1002, LTA = 50 µg/mL lipotheichoic acid from Staphylococcus aureus (n = 6/group). Cell cultures in Control group received none of the treatments.

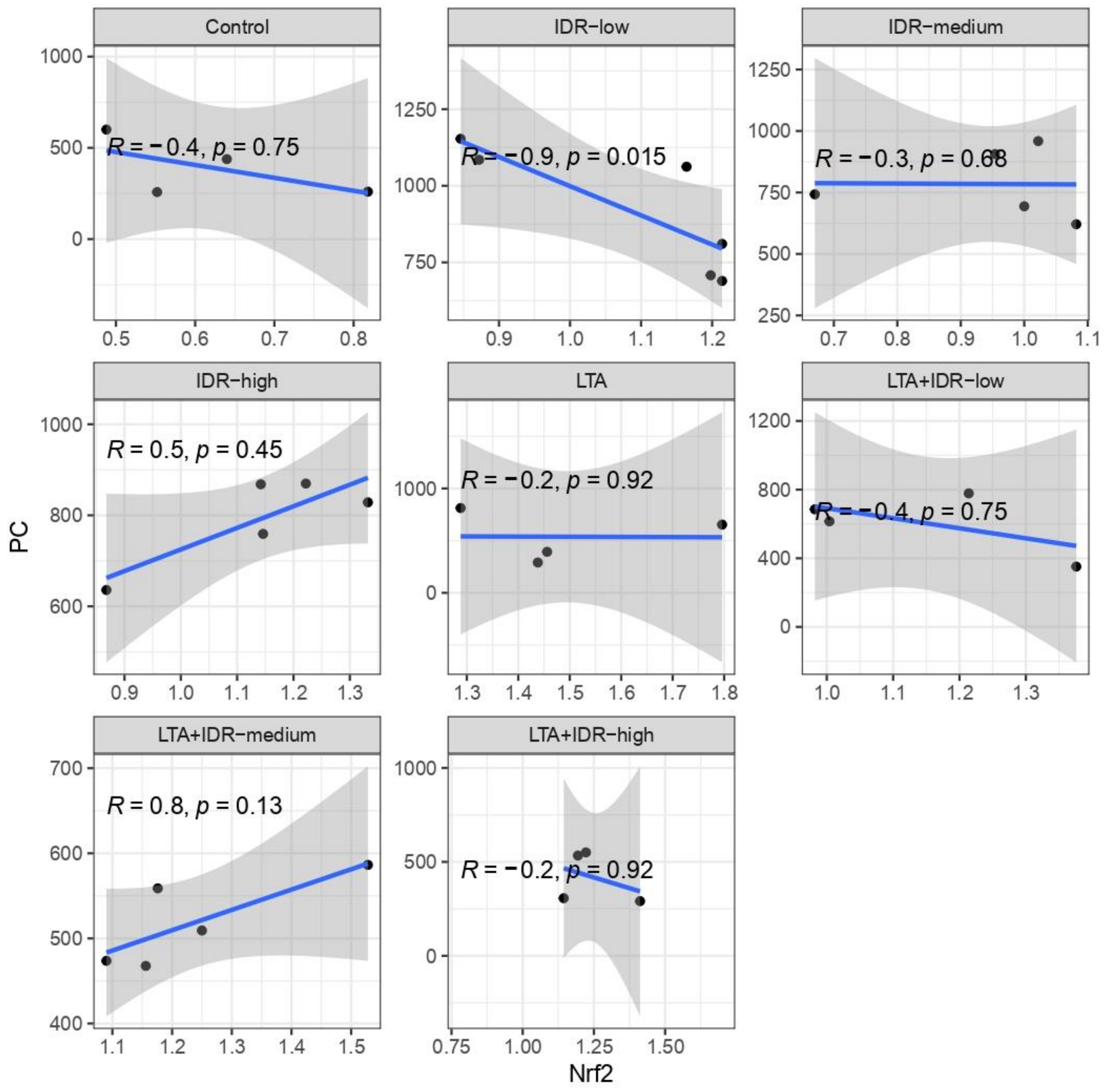

<u>Supplementary Figure 13:</u> Scatterplot showing the association between Nrf2 and Protein Carbonyl (PC) values for each treatment group.

Linear regression lines (with 95% confidence band) and Spearman correlation coefficients with p-values are displayed on each subplot for every treatment group. IDR-low = 10 µg/mL IDR-1002, IDR-medium = 30 µg/mL IDR-1002, IDR-high = 90 µg/mL IDR-1002, LTA = 50 µg/mL lipotheichoic acid from Staphylococcus aureus (n = 6/group). Cell cultures in Control group received none of the treatments.
