# Supplementary material for: Modulation of the immune response by the host defense peptide IDR-1002 in chicken hepatic cell culture
Source: Sci Rep. 2023 Sep 4;13:14530. doi: 10.1038/s41598-023-41707-z (PMC10477227; doi:10.1038/s41598-023-41707-z)
Supplement: Supplementary file 4 — Supplementary Legends. [file 41598_2023_41707_MOESM4_ESM.docx]

**Supplementary information file 1:** **File containing dataset of the present study**. Each row corresponds to one sample. Columns indicate the treatment group of the sample (column Treatment), unique identifier associated with it (column Sample ID), the remaining columns show the measured values of the parameter indicated in the header.

**Supplementary information file 2:** **File containing p-values for the pairwise Wilcoxon signed rank tests of the present study**. Rows corresponding to individual comparisons of treatment groups defined in the first two columns. Following columns indicate the p-values for each parameter defined in the header. P values which are less than 0.05 are indicated in bold.

**Supplementary information file 3: File containing supplementary heatmap and correlation plots**.
